# Brain metastatic outgrowth and osimertinib resistance are potentiated by RhoA in EGFR-mutant lung cancer

**DOI:** 10.1038/s41467-022-34889-z

**Published:** 2022-12-12

**Authors:** Sally J. Adua, Anna Arnal-Estapé, Minghui Zhao, Bowen Qi, Zongzhi Z. Liu, Carolyn Kravitz, Heather Hulme, Nicole Strittmatter, Francesc López-Giráldez, Sampada Chande, Alexandra E. Albert, Mary-Ann Melnick, Bomiao Hu, Katerina Politi, Veronica Chiang, Nicola Colclough, Richard J. A. Goodwin, Darren Cross, Paul Smith, Don X. Nguyen

**Affiliations:** 1 Department of Pathology, Yale University School of Medicine, New Haven, CT USA; 2 Yale Cancer Center, Yale University School of Medicine, New Haven, CT USA; 3 Imaging and Data Analytics, Clinical Pharmacology and Safety Sciences, AstraZeneca, Cambridge, UK; 4 Yale Center for Genome Analysis, Yale University School of Medicine, New Haven, CT USA; 5 Department of Cell Biology, Yale School of Medicine, New Haven, CT USA; 6 Department of Medicine (Medical Oncology), Yale University School of Medicine, New Haven, CT USA; 7 Department of Neurosurgery, Yale University School of Medicine, New Haven, CT USA; 8 DMPK, Early Oncology TDE, AstraZeneca, Cambridge, UK; 9 Global Oncology Medical Affairs, AstraZeneca, Cambridge, UK; 10 Bioscience, Early Oncology TDE, AstraZeneca, Cambridge, UK

**Keywords:** Metastasis, Cancer therapy, Cancer microenvironment

## Abstract

The brain is a major sanctuary site for metastatic cancer cells that evade systemic therapies. Through pre-clinical pharmacological, biological, and molecular studies, we characterize the functional link between drug resistance and central nervous system (CNS) relapse in Epidermal Growth Factor Receptor- (EGFR-) mutant non-small cell lung cancer, which can progress in the brain when treated with the CNS-penetrant EGFR inhibitor osimertinib. Despite widespread osimertinib distribution in vivo, the brain microvascular tumor microenvironment (TME) is associated with the persistence of malignant cell sub-populations, which are poised to proliferate in the brain as osimertinib-resistant lesions over time. Cellular and molecular features of this poised state are regulated through a Ras homolog family member A (RhoA) and Serum Responsive Factor (SRF) gene expression program. RhoA potentiates the outgrowth of disseminated tumor cells on osimertinib treatment, preferentially in response to extracellular laminin and in the brain. Thus, we identify pre-existing and adaptive features of metastatic and drug-resistant cancer cells, which are enhanced by RhoA/SRF signaling and the brain TME during the evolution of osimertinib-resistant disease.

## Abstract (web summary)

While EGFR-targeted therapies have clinical benefit, drug-resistant brain metastases present a major obstacle. Here, the authors identify a genetic signature in brain metastatic lesions associated with osimertinib resistance and find RhoA to have an important role in the resulting phenotype.

## Introduction

Up to 40–50% of lung cancer patients will develop metastasis in the central nervous system (CNS) over the course of their disease. The brain has long been considered a sanctuary site based on the limited penetrance of most systemic drugs across the blood-brain barrier (BBB). Therefore, historically, patients with brain metastases had few therapeutic options beyond radiation. Outcomes for patients treated with new systemic therapies are improving, but the incidence of brain metastasis remains a clinical challenge, and lung cancer patients with CNS metastases eventually have a worse prognosis than those without^1,2^.

Non-small cell lung cancer (NSCLC) is the most frequently diagnosed lung cancer. NSCLC expressing activating mutations in the Epidermal Growth Factor Receptor (EGFR) often develop brain metastases concomitant or subsequent to disease elsewhere in the body^3–5^. Many activating mutations in *EGFR* confer sensitivity of NSCLC tumors to specific tyrosine kinase inhibitors (TKIs), but drug resistance inevitably arises^6^. In the case of clinical EGFR-mutant brain metastases, similar rates of response in the brain and in the body are reported following initial treatment with TKI therapies^7^. Yet, it is not uncommon to subsequently see relapse in the brain discordant with control of extracranial sites, and the incidence of brain metastasis has been documented to increase in patients treated with first-generation TKIs^8–10^.

The third-generation EGFR TKI osimertinib was developed to inhibit the activity of drug resistance gatekeeper mutations and other activating EGFR-mutant receptors while sparing wild-type EGFR. Importantly, osimertinib also has improved penetrance across the intact BBB relative to other TKIs^11,12^, and it has become the standard of care for EGFR-mutant metastatic NSCLC. When administered in patients with early-stage EGFR-mutant NSCLC, osimertinib reduces the incidence of CNS metastases^13^. Osimertinib can also reduce the risk of CNS progression in advanced stage EGFR-mutant NSCLCs^14,15^. However, a proportion of patients still progress due to CNS disease in the second-line or front-line settings. Our understanding of the mechanisms of CNS progression following effective treatment responses in the brain remains incomplete.

In this work, we examine the functional links between drug distribution, molecular properties intrinsic to tumor cells, and the tumor microenvironment (TME) in brain metastases from EGFR-mutant NSCLC. We find that a gene expression profile regulated through RhoA and SRF potentiates metastatic EGFR-mutant NSCLC cells to persist and outgrow preferentially in response to the brain microvascular TME.

## Results

### Exposure to the brain TME promotes osimertinib persistence and resistance

**Fig. 1:**
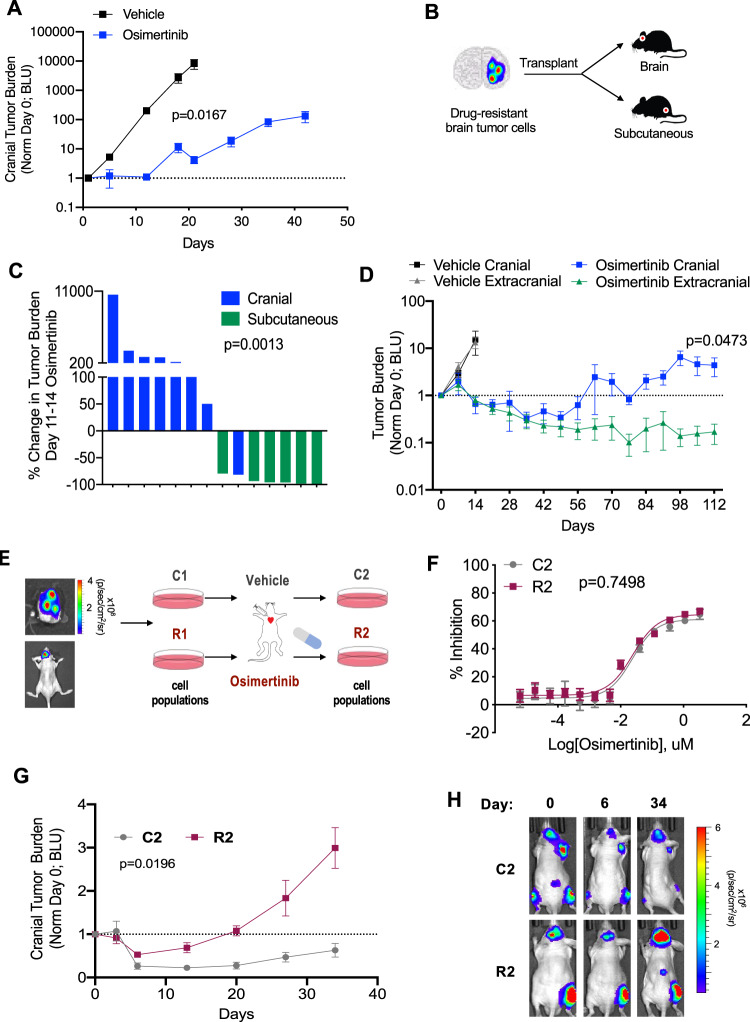
Exposure to the brain TME promotes osimertinib persistence and resistance. **A** H1975 cells were injected into the cranium of mice and then treated with vehicle or osimertinib three days later. Tumor burden was then measured by bioluminescence imaging (BLI) and bioluminescent units (BLU) plotted over time. *N* = 3 animals for vehicle, and *N* = 7 animals for osimertinib. Data presented as mean values +/− SEM. *P*-value calculated based on area under the curve (AUC) by Mann–Whitney (two-sided). **B** Experimental design in which osimertinib-resistant H1975 brain tumor cells were re-transplanted directly into the brains or hind flanks of treatment-naïve mice. **C** Waterfall plot of data from the experiment in **B**. Shown are % tumor burden changes of transplanted cells from Day 0 to Day 10–14 of treatment with osimertinib. Cranial tumor burden is determined by brain BLI and subcutaneous tumor burden is determined by flank tumor volume. *N* = 8 mice with cranial transplant, and *N* = 6 mice with subcutaneous transplant. *P*-value calculated by Mann–Whitney (two-sided). **D** PC9-BrM4 cells were injected into the arterial circulation of mice. Animals were then treated with vehicle or osimertinib after 23–51 days, and cranial and extracranial metastasis growth from the time of treatment were measured. For each animal, tumor burden is determined by BLI at a given timepoint and is normalized to BLI at Day 0 of treatment. *N* = 11 animals per group. Data presented as mean values +/− SEM. *P*-value calculated based on AUC by Mann–Whitney (two-sided). **E** Representative animal from (D) with confirmed brain parenchymal metastasis. Depicted is the subsequent isolation of C2 and R2 cell populations from brain metastases after 2 rounds of in vivo selection under vehicle or osimertinib treatment. **F** IC_50_ of osimertinib in C2 and R2 cells was calculated after 72 h of treatment in vitro. *N* = 3 samples per group. Data is representative of 2 independent experiments. Data presented as mean values +/− SEM. *P*-value calculated by *t*-test (two-sided). **G** Cranial tumor growth of mice with brain metastases treated with osimertinib was measured and plotted as in **D**. *N* = 4 animals for C2, and *N* = 15 animals for R2. Data presented as mean values +/− SEM. *P*-value calculated based on AUC by Mann–Whitney (two-sided). **H** Representative images from **G** at Day 0, 6, and 34 of osimertinib treatment.

To study the causes of osimertinib resistance in the brain, we first tested the in vivo metastatic response of the commonly utilized H1975 cell line, which carries the *EGFR* L858R and T790M mutations. These cells are sensitive to osimertinib when treated in vitro or as tumors grown in the flanks of mice^16^. We directly injected H1975 cells into the brain parenchyma (cortex) of mice, confirmed the presence of growing cranial tumors by bioluminescent imaging, and then treated animals with either vehicle or osimertinib at 25 mg/kg, which achieves an exposure equivalent to the clinical, 80 mg dose in humans^11,12,16^. While osimertinib significantly controlled brain metastatic progression compared to the vehicle control, brain tumors ultimately progressed in the presence of osimertinib (Fig. 1A and Supplementary Fig. 1A). We then collected GFP-positive tumor cells from the brains of mice with brain metastases that were growing on treatment and re-transplanted these cells directly into the brains or flanks of treatment-naïve recipient mice (Fig. 1B). In a head-to-head comparison, osimertinib caused regression of subcutaneous tumors while the majority of intracranial tumors generated by the same cells progressed on treatment as defined by RECIST-like criteria^17^ (Fig. 1C).

Next, we tested brain metastasis responses to osimertinib in an independent setting that also recapitulates concomitant systemic disease, by using the PC9-BrM4 cell line model, which carries *EGFR exon 19 del*. PC9-BrM4 cells were isolated from brain metastases formed in mice after four rounds of arterial injection (in vivo selection) of the parental PC9 cells carrying the *EGFR exon 19 del* mutation^18^. 23 days after injection of PC9-BrM4 cells into arterial circulation, most animals form cranial and extracranial tumors. Mice with disseminated disease were then treated with either vehicle or osimertinib at the clinically relevant dose of 25 mg/kg. Over the first 35 days of treatment, cranial and extracranial tumor burdens were significantly reduced in osimertinib-treated animals (Fig. 1D and Supplementary Fig. 1B). However, over 112 days of treatment, tumors eventually recurred, preferentially in the cranium, which we confirmed to be brain parenchymal metastases (Fig. 1D, E).

Given the latency of the acquired resistance phenotype in this model, we isolated tumor cells, termed PC9-BrM4-R1, from drug-resistant brain tumors. For a controlled comparison, we also isolated tumor cells, termed PC9-BrM4-C1, from brain metastases that had been treated with vehicle in parallel. PC9-BrM4-C1 and PC9-BrM4-R1 cells were then subjected to an additional round of in vivo selection with vehicle or osimertinib treatemnt, respectively, yielding PC9-BrM4-C2 (herein referred to as C2) and PC9-BrM4-R2 (herein referred to as R2) cells (Fig. 1E). When directly compared to passaged-matched C2 cells in vitro, R2 cells were equally sensitive to osimertinib (Fig. 1F). When injected into the arterial circulation of mice (intracardiac injection), R2 cells had a modestly higher brain metastatic capacity than C2 cells (Supplementary Fig. 1C). To compare in vivo drug responses, we treated C2 and R2 injected animals, 36 and 29 days, respectively, after intracardiac tumor cell injection. Interestingly, animals with either C2 or R2 metastases initially displayed a similar depth of response to osimertinib treatment within the first week of treatment (Fig. 1G, H). However, by day 34 of treatment, the majority of R2 mice manifested overt drug resistance, with a tumor burden that was at least 10x greater than the tumor burden at the time of maximal osimertinib response in a given animal (Fig. 1G, H). Conversely, the C2 injected mice had more durable responses and stable residual CNS disease with a tumor burden that was less than the cranial tumor burden on Day 0 of treatment (Fig. 1G, H). Over the course of our experiments, the control of extracranial metastases, which were mostly in the bones/hindlimbs (Fig. 1H), was more durable than the control of cranial metastases in all groups, and the R2 extracranial tumors eventually showed a latent and modest increase in growth when compared to the C2 extracranial tumors (Supplementary Fig. 1D). Further analysis of individual animals confirms that, irrespective of tumor burden before treatment, R2 brain metastatic growth was initially inhibited by osimertinib (Supplementary Fig. 1E, F) consistent with the fact that R2 cells are still intrinsically responsive to osimertinib treatment (see Fig. 1F) but are poised for adaptive resistance at later timepoints in vivo.

We conclude that the capacity of NSCLC cells for drug resistance is linked, in part, to brain metastatic potential and that the brain TME reduces the durability of osimertinib response.

### Long-term osimertinib resistance in the brain is independent of drug distribution or lack of target inhibition

**Fig. 2:**
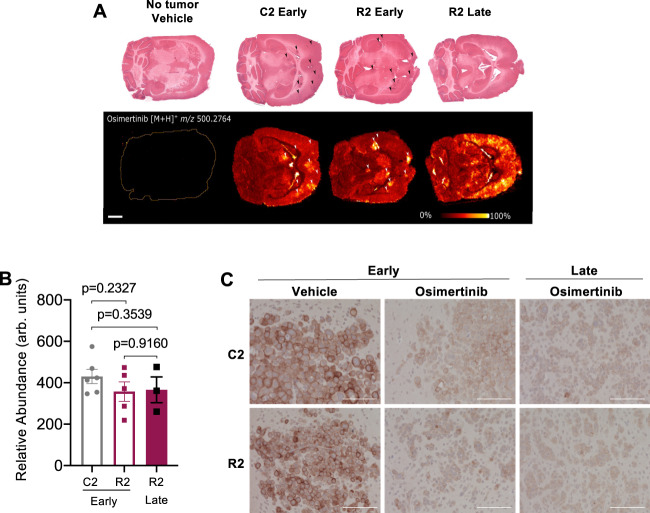
Long-term osimertinib resistance in the brain is independent of drug distribution or lack of target inhibition. **A** Representative H&E images (top) and MS images (bottom) of adjacent sections of brains harvested from mice with established C2 and R2 brain metastases treated with a single dose of osimertinib (“early”), or R2 treated with osimertinib continuously for 59 days (“late”). MS images are shown for osimertinib recorded as [M + H]^+^ at *m/z* 500.2764. MS images are also shown for a control brain, without osimertinib treatment. Arrows in C2 and R2 “early” images show small areas of metastases, while metastatic cells in R2 “late” were widely dispersed in the cortex. Scale bar indicates 2 mm. Spatial resolution of MSI experiments is 100 µm. **B** Relative abundance of osimertinib (measured in arbitrary units) in tumor tissue. *N* = 6 animals for C2 early, *N* = 5 animals for R2 early, and *N* = 3 animals for R2 late. Each point on the plot represents the average abundance for each mouse. All brains were collected 2 h after the last dose of osimertinib. Data presented as mean values +/− SEM. *P*-value calculated by *t*-test (two-sided). **C** pEGFR immunostaining of C2 and R2 brain metastases treated with vehicle or osimertinib for 3 days (“early”) or 37–43 days (“late”). Brains were collected 6 h after the last osimertinib dose. Scale bar indicates 100 μm. A representative image of one experiment is presented. Early: C2-vehicle (19 tumors/2 mice), C2-osimertinib (22 tumors/2 mice), R2-vehicle (21 tumors/2 mice), R2-osimertinib (24 tumors/2 mice), Late: C2-osimertinib (44 tumors/3 mice), R2-osimertinib (18 tumors /1 mouse).

In brain metastases, the limited depth and lack of durable response to systemic therapies have been attributed to poor penetration of drugs across the BBB. Although osimertinib is brain penetrant, it is possible that, over prolonged treatment periods, increased drug efflux or heterogenous drug distribution could lead to sub-optimal inhibition of EGFR signaling and partial responses. To investigate this possibility, we performed desorption electrospray ionization mass spectrometry imaging (DESI-MSI)^19^ using brain tissue containing metastases formed by C2 or R2 cells, after one day of 25 mg/kg osimertinib treatment. We also collected brain tissue from R2 drug-resistant mice after 59 days of continuous treatment. As previously reported^12^, osimertinib was widely distributed across normal and tumor-bearing regions of the brain (Fig. 2A). Slightly higher levels of osimertinib can be seen in brain lesions likely reflecting BBB leakage (Fig. 2A). However, there were no significant differences in the concentration of osimertinib in C2 and R2 brain metastases (Fig. 2B). Finally, immunohistochemical analysis of pEGFR in vivo shows that EGFR activity is decreased by osimertinib in all groups at all timepoints (Fig. 2C), confirming that acquired resistance in the brain is not due to limited drug exposure and lack of target inhibition at relevant doses.

### Molecular features of metastasis and osimertinib resistance are co-expressed in pre-existing NSCLC cell populations

**Fig. 3:**
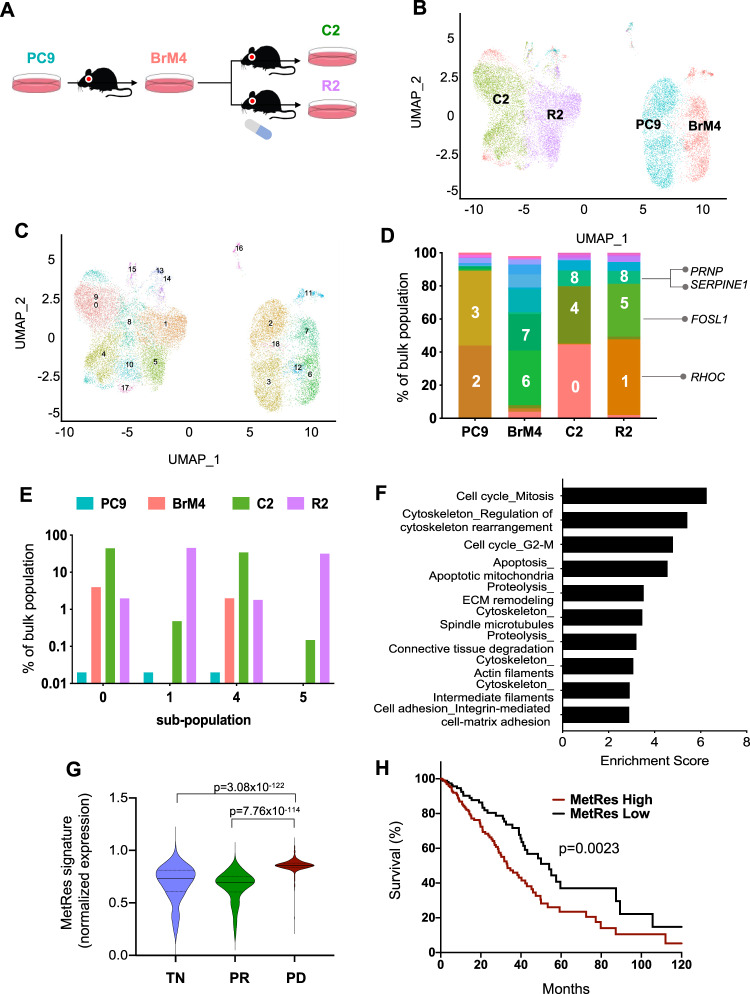
Molecular features of metastasis and drug resistance are co-expressed in pre-existing NSCLC cell populations. **A** Scheme of in vivo brain metastatic selection yielding PC9-BrM4, C2, and R2 bulk cell populations from the parental PC9 line. **B** scRNA-seq Uniform Manifold Approximation and Projection (UMAP) analysis of ~6000 cells from each of the bulk cell populations in **A**. **C** Same cells in **B** were re-colored based on distinct cell sub-populations as defined by UMAP analysis. **D** Stacked graph indicating the % of the sub-populations identified in **C** within the parental PC9, PC9-BrM4, C2, and R2 bulk populations. **E** Bar graph (log- scaled) plotting the % of C2 or R2 enriched sub-populations across the PC9, PC9-BrM4, C2, and R2 bulk populations. **F** Pathways most significantly upregulated in R2 enriched cell sub-populations (1, 5, and 8) compared to the C2 cells based on scRNA-seq analysis. Enrichment score is calculated by Metacore and plotted as –log_10_(*P*-value). **G** Differential mean expression of the dual Metastasis-Resistance (MetRes) signature is shown as a violin plot for single tumor cells collected from EGFR-mutant NSCLC patients (*N* = 20 patients)^24^. TN = treatment-naïve patients (*N* = 457 cells). PR = Patients with partial response after systemic therapy (*N* = 557 cells). PD = patients with progressive disease after systemic therapy (*N* = 1088 cells). *P*-value calculated by Welch *t*-test (two-sided). **H** Human LUADs from TCGA (*N* = 449) were classified as “high” or “low” based on whether expression of the MetRes signature was above or below the median, respectively. Kaplan–Meier curves were generated for the incidence of death of “high” vs. “low” groups. *P*-value calculated by log-rank test. The MetRes signature was generated for each sample (single cells in **G**; bulk tumors in **H**) by calculating the average expression of all MetRes genes identified in Supplementary Data 2.

Two observations suggested that adaptive drug resistance in our brain metastasis models could be attributed to selectable traits of cancer cells under drug treatment in vivo. First, the magnitude of the resistant capacity of metastatic cells seemed to increase with their subsequent passaging in vivo (Supplementary Fig. 2A). Second, despite the passaging of these cells in vitro without drug, the differential in vivo response of R2 brain metastases was reproducible across four independent experiments (Supplementary Fig. 2B). To identify features of tumor cell sub-populations that are specifically linked to drug resistance and/or brain metastasis, we performed single-cell RNA sequencing (scRNA-seq) on the PC9 parental, PC9-BrM4, C2, and R2 bulk cell populations cultured in vitro in the absence of osimertinib (Fig. 3A). The uniform manifold approximation and projection (UMAP) generated by scRNA-seq show that cells from PC9 and PC9-BrM4 bulk populations cluster together while cells from the C2 and R2 samples cluster together (Fig. 3B), consistent with the increased selective pressure imposed on the C2 and R2 cells relative to the parental PC9 and PC9-BrM4 bulk populations. scRNA-seq revealed 19 different cell sub-populations (labeled 0–18), some of which were differentially distributed across the bulk PC9 populations (Fig. 3C).

Next, we plotted the UMAP for each individual sample (Supplementary Fig. 2C) and also quantified the percentage of distinct sub-populations across all samples (Fig. 3D). We then scrutinized the frequency of sub-populations that are prevalent in C2 and/or R2 samples, considering that C2 cells were selected for aggressive brain metastatic competence in the absence of drug, while R2 cells were selected in parallel for both brain metastatic competence and drug resistance. First, we noted that sub-populations that dominate C2 or R2 samples pre-exist at lower frequency in the parental PC9 and PC9-BrM4 cells (Fig. 3E). Second, the frequency of these sub-populations was higher in both C2 and R2 cells, albeit at variable frequencies. For example, some sub-populations were either most prevalent in C2 cells (e.g., 0 and 4), R2 cells (e.g., 1 and 5), or were equally enriched in both samples (e.g., 8) (Fig. 3D, E). Hence, the selection for osimertinib-resistant cells may be a collateral effect of metastatic progression in the absence of drug, while drug treatment in vivo may further select for resistant sub-populations with enhanced capacity for brain metastatic outgrowth.

Ultimately, our scRNA-seq data indicate that pre-existing cell sub-populations that are either metastatic or poised for osimertinib resistance share common molecular features. Genes highly expressed in R2-enriched cell sub-populations include regulators of cytoskeletal re-modeling (*Ras homolog gene family, member C; RHOC*)^20^, extracellular matrix (ECM) turnover (*Plasminogen activator inhibitor-1*; *SERPINE1*)^21^, mitosis/transcription (*Fos-related antigen 1*; *FOSL1*)^22^, and neuronal synapse (*Prion protein; PRNP*)^23^ (Fig. 3D, F). Some, but not all, of these genes were also increased in C2 sub-populations, which are metastatic but not overtly TKI resistant (Supplementary Data 1). We refer to these putative dual markers of metastasis and drug resistance as Metastasis-Resistance (MetRes) genes (255 genes, Supplementary Data 2). The identification of MetRes features in a smaller fraction of treatment-naïve and poorly metastatic cells (e.g., PC9 parental) suggests that their expression in extracranial or early-stage tumor cells could be associated with drug resistance or poor outcome. A prior study isolated NSCLC cells from re-biopsies (mostly lung tissue) of human patients with either treatment-naïve (TN), partial responses (PR), or progressive disease (PD) following various systemic therapies including TKIs^24^. We calculated MetRes gene signature expression across this NSCLC cohort. Consistently, MetRes features were enriched in cancer cells from EGFR-mutant NSCLC patients with progressive disease (Fig. 3G). This correlation was also significant when analyzing human NSCLCs with other driver mutations (e.g., ALK-mutant) or when pooling all available cases (Supplementary Fig. 2D, E). In addition, high expression of the MetRes signature in lung adenocarcinomas from TCGA correlated with poor patient survival (Fig. 3H).

### Colonization of the brain TME increases cytoskeletal and ECM signaling genes in osimertinib-resistant metastatic cells

As the acquired resistance in our models preferentially occurs in the brain, we also analyzed the molecular features of brain metastatic cells under treatment in vivo. First, we collected brain metastasis tissue from C2 and R2 cells after three days of either vehicle or osimertinib treatment (referred to as “early” timepoint) (Supplementary Fig. 3A). We then collected drug-resistant R2 brain metastasis tissue when metastatic burden was at least 10x greater than metastatic burden at the time of maximal osimertinib response in a given animal (referred to as “late” timepoint) (Supplementary Fig. 3A, B). We also collected time-matched late samples from osimertinib-treated animals with residual C2 tumors (Supplementary Fig. 3A). Additionally, we collected C2 and R2 cells in vitro treated with either vehicle or osimertinib for 24 h, when there is no significant difference in the growth of C2 and R2 cells (Supplementary Fig. 3C). For transcriptomic analysis, we used a brain metastasis xenograft RNA-sequencing (BMX-seq) pipeline that we previously optimized to distinguish tumor from stromal gene expression in intact brain metastatic tissue^25^. Similarly, we collected C2 and R2 cells in vitro and R2 cells from late timepoints in vivo for whole-exome sequencing (WES).

WES identified only five non-synonymous variants (four missense mutations and one deletion) that were highly expressed in R2 cells when compared to C2 cells (Supplementary Table 1). None of these variants occur in *EGFR*. Rare Exome Variant Ensemble Learner (REVEL) scores for the missense mutations are all below 0.5, suggesting low pathogenicity of these variants^26^. The in-frame deletion occurs in a highly repetitive region of *RIN3* that is highly frequent in the normal human population both in the heterozygous and homozygous form^27^, suggesting that it is not a pathogenic driver. By contrast, BMX-seq revealed numerous changes in tumor cell-specific gene expression patterns, which distinguish C2 and R2 samples by context (in vivo vs. in vitro) or treatment (osimertinib vs. vehicle) (Supplementary Fig. 3D). The number of differentially-expressed tumor genes was higher when comparing R2 to C2 samples in vivo at early timepoints (when tumor burden is similar) whereas the same comparison using in vitro samples yielded fewer differences (Supplementary Fig. 3E).

**Fig. 4:**
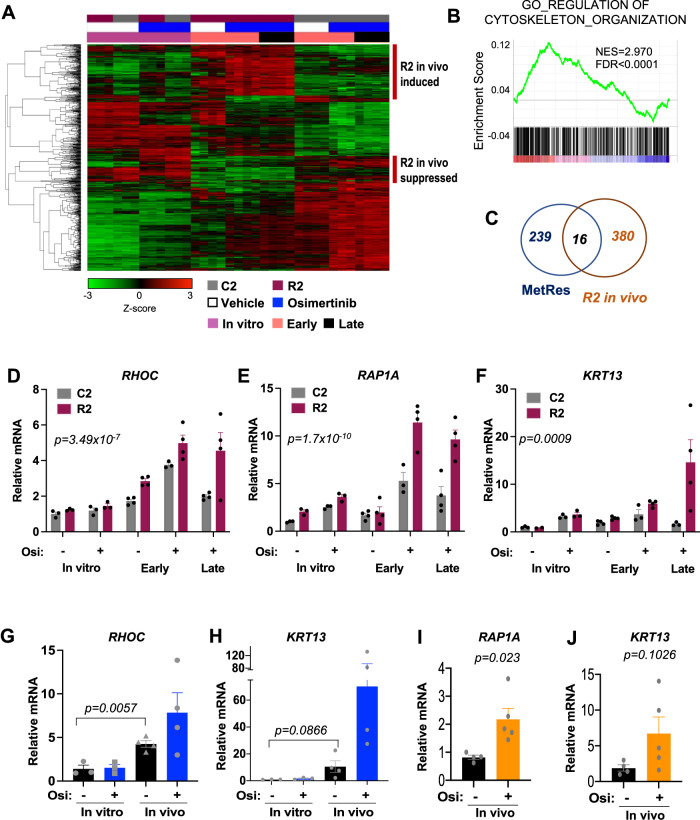
Features of brain metastatic and drug-resistant cancer cells are enhanced in vivo. **A** “Early” whole brain tissues were collected after three days of continuous treatment and “late” brain tissues 34–58 days after osimertinib treatment. All samples were collected 6 h after the last dose of osimertinib. Heatmap depicts hierarchical clustering of the 847 tumor cell-specific genes that are significantly differentially expressed between the R2 and C2 brain metastases in both “early” and “late” in vivo samples. Red vertical bars denote genes that are preferentially induced or repressed in R2 cells when compared to C2 cells in vivo. *N* = 3 (in vitro, C2 early/vehicle), *N* = 4 (R2 early and late; C2 early/osi and late). **B** Gene set enrichment analysis result for GO_REGULATION_OF_CYTOSKELETON_ORGANIZATION comparing tumor cell-specific genes expression in early R2 to early C2 in vivo samples. **C** Venn diagram depicting the intersection between genes identified in **A** and the MetRes signature. **D**–**F** Relative expression of the indicted genes was measured by species-specific qPCR and by comparing R2 vs. C2 samples in vitro and in vivo that were treated with vehicle or osimertinib. *N* = 3 (in vitro, C2 early/vehicle), *N* = 4 (R2 early and late; C2 early/osi and late). Data presented as mean values +/− SEM. *P*-value calculated by ANOVA. **G**, **H** Relative expression of the indicted genes was measured by species-specific qPCR in H1975 tumor cells grown in vitro or in vivo and treated with vehicle or osimertinib. *N* = 3 biological replicates for all conditions except in vivo vehicle *N* = 4. Data was normalized to *HPRT* and plotted with SEM. **I**–**J** Relative expression of the indicted genes was measured in the PDX model YU-006 as in **G**, **H**. *N* = 4 tumors for vehicle, and *N* = 5 tumors for osimertinib. For **G**–**J**, Data presented as mean values +/− SEM and *P*-values calculated by Welch’s *t*-test (two-sided).

We prioritized tumor genes that were differentially expressed at early in vivo timepoints and whose expression pattern was maintained in late samples (*n* = 847) (Supplementary Data 3). Hierarchical clustering of all samples confirms that these genes predominantly distinguish C2 and R2 metastatic samples in vivo and that this distinction was not strictly dependent on osimertinib treatment (Fig. 4A, e.g., R2 in vivo-specific genes annotated with red bar). When comparing gene response in vivo, there is significant enrichment of pathways related to the regulation of the cytoskeleton and ECM signaling in R2 cells (Fig. 4B). Notably, expression of epithelial to mesenchymal (EMT) genes or neuroendocrine enriched genes, both of which have been associated with TKI resistance, were not significantly altered in R2 cells in vitro or in vivo (Supplementary Fig. 3F). A subset of 16 in vivo upregulated gene alterations (e.g., *RHOC*) overlap with the MetRes signature identified by scRNA-seq in R2 sub-populations in vitro (Fig. 4C, D). Other genes in this category were also differentially expressed in bulk R2 cells, but their expression was further upregulated by osimertinib treatment in the brain. This included the cell adhesion GTPase *Ras-related protein 1 (RAP1A*)^28^ and the cytoskeleton filament gene* Keratin 13 (KRT13)*^29^ which were validated by species-specific quantitative polymerase chain reaction (qPCR) (Fig. 4E, F). We also found similar transcriptional responses to be induced by the brain or osimertinib treatment in vivo in the H1975 model or a patient-derived brain metastasis xenograft (YU-006; which was primarily maintained in vivo), with *RHOC* and *RAP1A* upregulation achieving significance where indicated (Fig. 4G–J).

Collectively, our molecular analysis indicates that tumor cells that are poised for osimertinib resistance and brain metastatic colonization are defined by cell-intrinsic transcriptional alterations associated with cytoskeletal and ECM re-modeling. Furthermore, the brain TME and osimertinib treatment can independently enhance the expression of some of these genes in vivo.

### Vascular phenotypes associated with osimertinib persistence and resistance in vivo

**Fig. 5:**
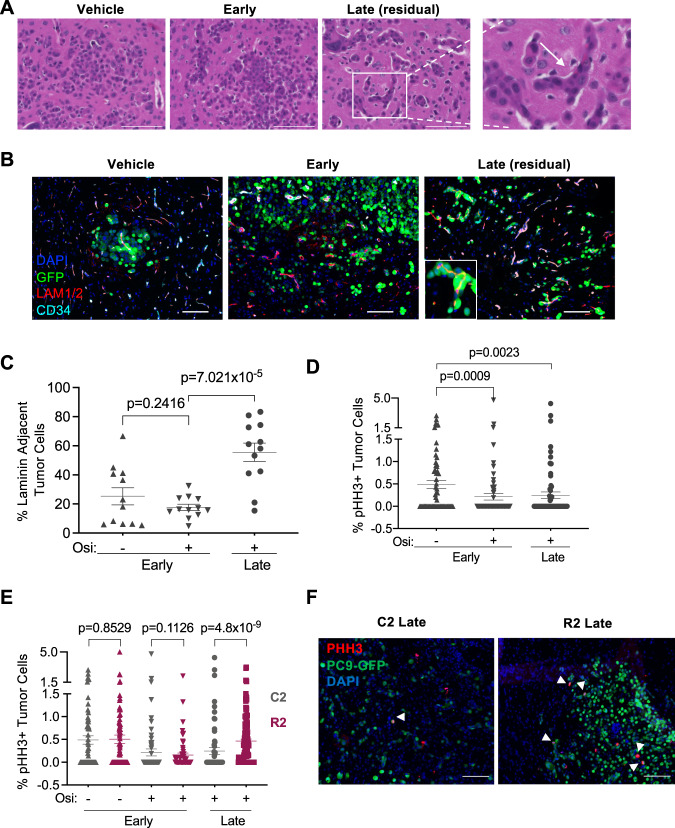
Re-modeling and co-option of the brain vasculature correlates with residual disease. **A** Representative images of hematoxylin-eosin- (H&E-) stained brain tissue sections from mice with C2 brain metastases treated with vehicle or osimertinib and collected at “Early” or “Late” timepoints as in Fig. 4A. Scale bar indicates 100 μm. Inset is an expanded view of Late sample. White arrow indicates brain perivascular residual tumor cells in osimertinib-treated animal. Representative images from Vehicle (4 tumors/2 mice), Early (4 tumors/2 mice), Late residual (8 tumors/3 mice). **B** Representative images of C2 brain metastasis tissue (N = 12 images from 3 mice) collected as in **A** and subjected to immunofluorescent (IF) staining for nuclei (DAPI; blue), tumor cells (GFP; green), laminin (red), and vasculature (CD34; turquoise). Scale bar indicates 100 μm. **C** The number of GFP-positive C2 tumor cells adjacent to laminin-positive micro-vessels were manually counted from images captured as in **B**, graphed as a percentage of total tumor cells, and presented as mean values +/− SEM. *N* = 12 images of 3 animals per group. *P*-values calculated by Welch’s *t*-test (two-sided). **D**, **E** Percentage of phospho-histone H3 (pHH3)-positive tumor cells was quantified as in **C**. C2 (gray): vehicle early, *N* = 8; osimertinib early, *N* = 73; osimertinib late, *N* = 82. R2 (maroon): vehicle early *N* = 76, osimertinib early *N* = 69, osimertinib late *N* = 157, where each *N* = an independent area of different tumors. Images are from 3 animals per group. Data is presented as mean values +/− SEM. *P*-values for IF quantification calculated by Mann–Whitney (two-sided). **F** Representative IF images (from **D**–**E**) for pHH3 in the indicated samples from mouse brain with metastasis. Arrows denote pHH3-positive tumor cells.

We next evaluated which features of the brain TME associate with osimertinib response, persistence, and resistance in vivo. Once disseminated tumor cells (DTCs) extravasate into the brain parenchyma, the initial survival and outgrowth of micrometastases requires either the co-option of exiting microvasculature or vascular re-modeling by DTCs^30^. When treated with osimertinib, established C2 brain tumors regress but there are residual tumor cells in the brain as confirmed by histological staining (Fig. 5A). Residual brain metastatic cells were also detected in the H1975 and PDX-YU-006 models after 41 and 40 days respectively of osimertinib treatment. In these models, long-term osimertinib- treated lesions displayed an increase in CD34-positive-associated laminin (Supplementary Fig. 4A, B), indicative of vascular basement membrane re-modeling^31^. Additionally, residual C2 cancer cells manifested as perivascular lesions (Fig. 5A) resembling miliary tumors, which are clinically detected within the subpial space of humans with CNS metastases^32,33^.

The incidence of perivascular miliary metastasis is higher in patients with EGFR-mutant lung adenocarcinoma^34^, and it has been suggested that this clinicopathological pattern could be a reservoir of persistent cancer cells^32^. Also, persistent NSCLC cells in humans treated with TKIs may exist in a growth arrested or slow cycling state^24^. The percentage of residual C2 cells directly in contact with laminin-positive vessels increased with osimertinib treatment (Fig. 5B, C). This correlated with a decrease in phospho-histone H3 (pHH3)-positive proliferating C2 cells, and, at late timepoints, low levels of pHH3 were observed in residual cancer cells (Fig. 5D), suggesting that these are a drug persistent population. When comparing metastasis from R2 and C2 cells, we found no difference in the percentage of cleaved caspase-3-positive (Cl-casp3) apoptotic cells in the brain (Supplementary Fig. 4C). Likewise, the amount of R2 cells directly adjacent to laminin-positive blood vessels was similar to that of C2 cells in tumors treated with osimertinib (Supplementary Fig. 4D, E). However, at late timepoints, the amount of pHH3-positive R2 cells was increased (Fig. 5E, F), consistent with their higher expression of cell proliferative genes and ability to resume perivascular outgrowth after osimertinib treatment.

Thus, re-modeling and/or co-option of the brain microvasculature may initially contribute towards the persistence of DTCs under osimertinib treatment in vivo. Furthermore, brain metastatic cells that derive a proliferative advantage within this niche are poised to subsequently emerge as overtly resistant to TKI.

### Laminin and RhoA potentiate features of drug resistance and metastatic outgrowth

**Fig. 6:**
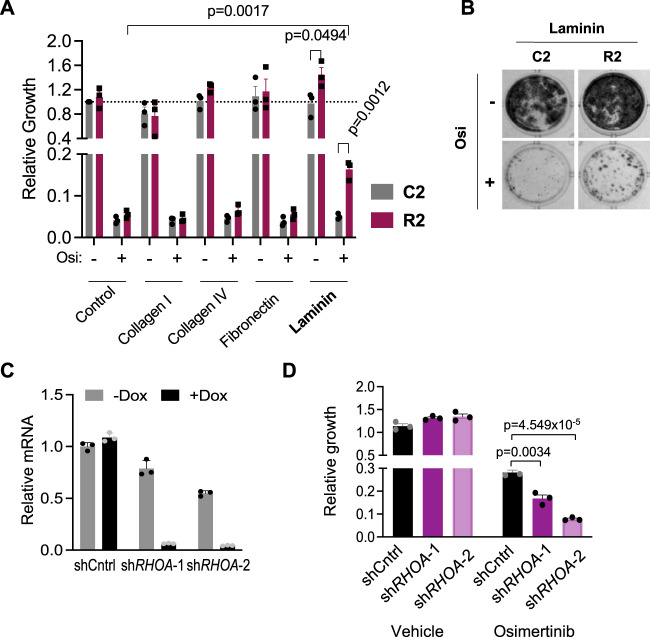
Laminin and RhoA promote the outgrowth of residual metastatic cells under osimertinib treatment in vitro. **A** C2 and R2 cells were cultured on standard plates (Control) or plates coated with the indicated ECM proteins and treated with vehicle or 160 nM osimertinib. Relative tumor cell outgrowth was measured by BLI 18–21 days after plating. All values are normalized to C2 vehicle-treated on control plate. *N* = 3 biological replicates. Data is presented as mean values +/− SEM. *P*-values calculated by *t*-test (two-sided). **B** Representative images of samples in **A** grown on laminin and stained with crystal violet. **C** R2 cells expressing doxycycline- (Dox-) inducible short hairpins RNAs (shRNAs) against a control sequence (shCntrl) or independent shRNAs targeting *RHOA* (sh*RHOA*−1 and sh*RHOA*−2) were cultured in the absence or presence of Dox. *RHOA* was measured by qPCR and normalized to *HPRT* expression. Data is presented as mean values +/− SD. A representative of two independent experiments is shown. **D** R2 cells with the indicated shRNAs were cultured in the presence of Dox on laminin-coated plates and treated with vehicle or osimertinib. Relative tumor cell growth was measured as in **A**. All values are normalized to R2 shCntrl vehicle-treated on control plates. *N* = 3. Data from a representative experiment (of 3 separate experiments) is presented as mean values +/− SEM. *P*-values calculated by *t*-test (two-sided).

In the normal CNS and brain tumors, blood vessels are the major source of extracellular matrix (ECM) and basement membrane (BM) proteins including laminin and collagen-IV, which can directly contribute to metastatic cell survival and outgrowth from dormancy^35,36^. To ascertain if ECM proteins support the emergence of osimertinib-resistant brain metastatic cells, C2 and R2 cells were cultured in the presence of growth factors on plates coated with fibronectin, collagen I, collagen-IV, or laminin and treated with vehicle or osimertinib for 21 days. The overall growth of C2 and R2 cells was reduced by osimertinib, irrespective of ECM conditions (Fig. 6A). However, laminin significantly increased long-term outgrowth of R2 cells compared to C2 cells, especially when treated with osimertinib (Fig. 6A, B). This is consistent with the observation that perivascular R2 cells can resume proliferation after a period of osimertinib-induced latency in vivo (see Fig. 5E). When grown on other ECM proteins, tumor cells also persist and undergo some morphological changes (Supplementary Fig. 5A), but these conditions did not increase the outgrowth of R2 cells relative to C2 cells (Fig. 6A and Supplementary Fig. 5B).

Genes encoding for the laminin-binding integrin receptor subunits *α6 and β1*^37^ were already expressed at high levels in both C2 and R2 cells (Supplementary Fig. 5C). In addition, other laminin-binding receptors (e.g., *ITGA2* and *ITGA3*) (Supplementary Data 2) and cytoskeleton re-modeling genes (Figs. 3F and 4B) were upregulated in sub-populations of R2 cells when compared to C2 cells. Many of these genes converge onto RhoGTPase^38^, suggesting that mechano-signaling could be regulating osimertinib resistance and brain metastasis. Interestingly, osimertinib treatment by itself increased the proportion of R2 and C2 cells with phosphorylated myosin light chain (pMLC) (Supplementary Fig. 5D, E), which is a marker of increased mechanotransduction via RhoGTPases^38^. Since there was no difference in pMLC between C2 and R2 cells, activation of mechano-signaling is an adaptive response of metastatic EGFR-mutant cancer cells as they are initially responding to osimertinib.

Of the different RhoGTPases, activated Ras Homolog Family Member A (RhoA) not only signals downstream of integrin receptors but also bidirectionally stabilizes their interaction with laminin, which is central for epithelial cell binding to the BM during mammalian development^39^. Accordingly, we first tested the requirement of RhoA in the metastatic drug-resistant R2 cells. Although *RHOA* was similarly expressed in C2 and R2 cells (Supplementary Fig. 6A), knockdown of *RHOA* using independent short hairpin sRNAs (shRNAs) reduced the capacity of R2 cells to outgrow on laminin after long-term osimertinib treatment (Fig. 6C, D). Knockdown of *RHOC*, another RhoGTPase^20^ that was elevated in R2 cells, did not have a significant effect, whereas knockdown of *Integrin* β1 (ITGB1)^37^, a requisite co-receptor for laminin-binding integrins, caused a reduction in metastatic cell outgrowth under the same conditions (Supplementary Fig. 6B–E).

**Fig. 7:**
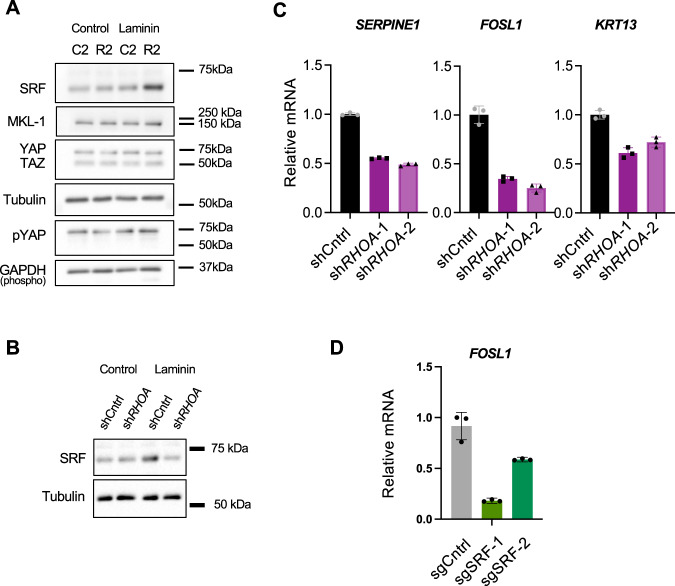
RhoA controls SRF protein levels and the expression of brain metastasis and drug resistance genes. **A** C2 or R2 cells were cultured over 18 days in the presence of osimertinib on control or laminin-coated plates. Lysates were then subjected to western blotting for the indicated proteins. A representative blot from two independent experiments is shown. **B** R2 cells expressing the indicated shRNAs were cultured as in **A**. Lysates were then subjected to western blotting for SRF or tubulin. A representative blot from two independent experiments is shown. **C** R2 cells expressing the indicated shRNAs were cultured on laminin-coated plates with Dox and serum starved for 12 h. Expression of *SERPINE1*, *FOSL1*, and *KRT13* were measured by qPCR, normalized to *HPRT* expression and data was plotted with SD. A representative of two independent experiments is shown. *N* = 3 technical replicates. **D** R2 cells expressing the indicated sgRNAs were cultured on laminin-coated plates and treated as in **B**. Expression of *FOSL1* were quantified as in **C**. *N* = 3 technical replicates. Data presented as mean +/− SD and representative from 2 independent experiments for sgSRF#2 and 3 independent experiments for sgSRF#1.

RhoA can regulate actin dynamics via the LIMK and cofilin pathway or integrate cytoskeletal re-modeling with growth factor signaling via the transcription factors Hippo/Yes-Associated protein (YAP), Myocardin-related transcription factor (MRTF), or Serum Response Factor (SRF)^40,41^. Levels of cofilin, phosphorylated cofilin, MRTF, YAP, and phosphorylated YAP were unchanged in R2 cells after they were cultured on laminin and treated for 18 days with osimertinib (Fig. 7A and Supplementary Fig. 7A). Alternatively, under the same conditions, SRF protein levels, but not *SRF* mRNA, was higher in R2 cells relative to C2 cells (Fig. 7A and Supplementary Fig. 7B). SRF protein was generally reduced by long-term osimertinib treatment, and this effect was mitigated by laminin (Supplementary Fig. 7C). Importantly, RhoA knockdown in R2 cells reduced SRF protein (Fig. 7B), indicating that RhoA is required for the overall maintenance of SRF in this context. Moreover, bypass receptor tyrosine kinase (RTK) signaling via ERK or AKT phosphorylation was not consistently affected by RhoA knockdown (Supplementary Fig. 7D).

Next, we tested if RhoA or SRF were required for downstream expression of certain MetRes and cytoskeleton regulatory genes identified by scRNA-seq or bulk BMX-seq. *RHOA* knockdown in R2 cells consistently reduced the expression of *SERPINE1, FOSL1, and KRT13* (Fig. 7C). Notably, FOSL1 is a direct genomic target of SRF in contractile fibroblasts^42^. Knockdown of SRF also reduced the expression of *FOSL1* in R2 cells (Fig. 7D) and diminished metastatic cell outgrowth on laminin after long-term osimertinib treatment (Supplementary Fig. 7E, F). Finally, *SERPINE1, FOSL, and KRT13* are upregulated in NSCLC cells isolated from human patients that progressed on systemic therapies vs. patients with treatment-naive or residual disease (Supplementary Fig. 7G, H)^24^. Altogether, these data demonstrate that RhoA, in part through SRF, potentiates the expression of genes linked to drug resistance in humans with EGFR-mutant NSCLC.

### Inhibition of RhoA reduces brain metastatic outgrowth and increases osimertinib durability in vivo

**Fig. 8:**
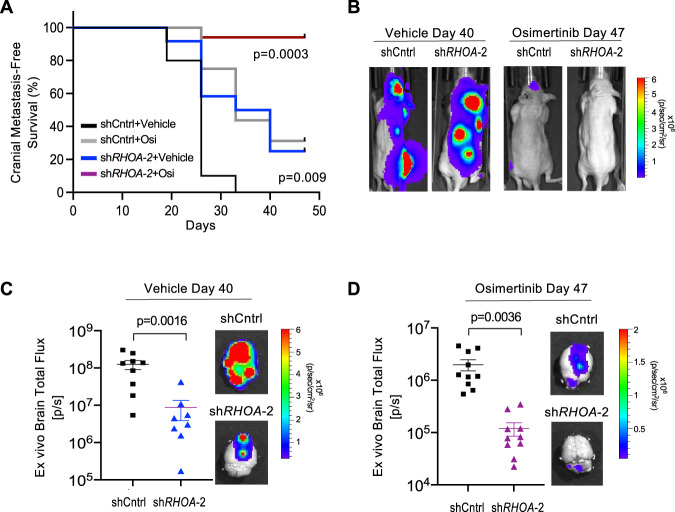
RhoA inhibition decreases brain metastatic outgrowth and osimertinib resistance in vivo. **A** Kaplan–Meier analysis of brain metastasis incidence following intracardiac injection of R2 shCntrl or R2 sh*RHOA*−2 cells into mice that were maintained on a Dox food diet and treated with either vehicle or osimertinib starting 12 days after injection. Brain metastasis incidence was detected by BLI. *N* = 10 animals for shCntrl/vehicle, *N* = 16 animals for shCntrl/osimertinib, *N* = 12 animals for sh*RHOA-2/*vehicle, and *N* = 17 animals for sh*RHOA-2*/osimertinib. *P*-values of *shRHOA* groups (compared to shCntrl+Vehicle) calculated by log-rank test. **B** Representative BLI images of animals with median cranial tumor burden from **A** for each group at the indicated timepoint. **C** Dot plot of ex vivo brain BLI from vehicle-treated R2 shCntrl and R2 shRHOA-2 mice harvested at Day 40. *N* = 9 animals for shCntrl, and *N* = 8 animals for sh*RHOA-2*. Data is presented as mean values +/− SEM. *P*-value calculated by Mann–Whitney (two-sided). **D** Dot plot of ex vivo brain BLI from osimertinib-treated R2 shCntrl and R2 sh*RHOA*−2 mice harvested at Day 47. *N* = 10 animals per group. Data is presented as mean values +/− SEM. *P*-value was calculated by Welch’s *t*-test (two-sided). For **C** and **D**, images of brains are from animals with median tumor BLI signal.

Osimertinib is approved for use as a front-line therapy in treatment-naïve patients and can delay but not completely prevent progression of late-stage CNS metastases. Thus, we tested whether RhoA inhibition could affect brain metastasis formation or the durability of osimertinib response in vivo. As osimertinib is also being used to treat patients that have yet to develop detectable metastases, we started dosing osimertinib in animals 12 days after the arterial injection of R2 cancer cells into mice. At this timepoint, most DTCs will have extravasated into the brain and display the earliest evidence of tumor cell survival^30^. R2 cells, which express the *EGFRdel19* mutation, eventually form brain metastases even when animals are treated and respond at this early stage (Supplementary Fig. 8A, B). RhoA knockdown or osimertinib treatment alone each decreased the incidence of brain metastasis as detected by longitudinal live animal imaging (Fig. 8A). Importantly, the combination of osimertinib with RhoA knockdown cooperatively reduced the incidence of brain metastases (Fig. 8A, B). Although the median progression-free survival time was not reached in this experiment, RhoA knockdown also decreased cranial progression in osimertinib-treated mice (*P* = 0.03). Ex vivo imaging of brains at endpoint (Day 40–47) revealed residual tumor cell signal in all samples tested (Fig. 8C, D), confirming that DTC dissemination into the brain was not affected in this experiment. However, the residual tumor burden of sh*RHOA* expressing lesions was significantly reduced relative to shCntrl tumors, particularly when combined with osimertinib treatment (Fig. 8D).

We also tested the effects of RhoA in the H1975 cells, which express *EGFR L858R and T790M* mutations and form metastasis in mice even when osimertinib treatment is started 2 days after cancer cell injection (Supplementary Fig. 8D, black and gray curves). Knockdown of RhoA in the more aggressive H1975 cells marginally reduced their ability to form detectable cranial metastasis in osimertinib-treated animals (Supplementary Fig. 8C, D), but nonetheless significantly delayed the progression of treated brain metastases (Supplementary Fig. 8E). Finally, in osimertinib-treated animals with either R2 or H1975 metastases, knockdown of RhoA had no additive effect on the incidence or progression of extracranial tumors (Supplementary Fig. 8F, G).

In summary, our findings reveal a role for RhoA-mediated signaling and gene expression during the outgrowth of residual EGFR-mutant cancer cells in the brain and progression of CNS metastases under osimertinib treatment.

## Discussion

The majority of EGFR-mutant NSCLC patients are diagnosed with advanced disease, and despite significant response when treated with osimertinib, a proportion of patients may still progress^14,15^. Several non-mutually exclusive mechanisms that limit osimertinib response have been identified in the context of systemic disease, with most of these directly attributed to genetic alterations in the tumor cells (e.g., subtype of EGFR mutation^43,44^ and other co-occurring mutations^45^) or adaptive molecular changes by cancer cells in response to drug alone. It is less clear how the TME can modify the depth and durability of osimertinib responses. Prior studies show that the improved ability of osimertinib to control CNS tumor growth correlates with its capacity to penetrate an intact BBB^12^. Nevertheless, despite initial osimertinib CNS activity, the development of brain metastatic drug resistance may still develop in animal models and humans.

In this pre-clinical study, we demonstrate that the brain is particularly hospitable for residual tumor cells, despite robust penetration of osimertinib and EGFR inhibition over periods of continuous treatment. Hence, the sanctuary nature of the brain is likely determined by factors that are independent of BBB integrity or drug efflux that can restrict drug penetration as observed with earlier generation TKIs. Certain intracellular signaling pathways have been linked to brain relapse and osimertinib resistance in EGFR-mutant NSCLC^46^. Importantly however, prior studies have yet to address which features of the brain TME influence the adaptive response and evolution of osimertinib-treated tumors in vivo. After debulking macrometastases with osimertinib, we found residual cancer cells that are either localized near the CNS microvasculature or are associated with a re-modeled vascular BM. These vascular phenotypes are reminiscent of what is observed during the early stages of treatment-naïve brain micrometastases and glioblastoma initiation^47^. Moreover, clinically, the appearance of residual perivascular lesions is akin to miliary brain metastases that persist in the Virchow-Robins space and may be more frequently observed in patients with EGFR-mutant NSCLC^32–34^. While many factors in the perivascular niche of the brain could influence treatment outcomes, we identified laminin as an important stromal factor, which limits the durability of response to osimertinib. In the brain at least, the endothelial BM is a predominant source of laminin^31^. Collectively, these findings suggest that metastatic cells that have adapted to the brain microvascular environment may be poised for survival in this laminin rich reservoir when they are under subsequent selective pressure from therapy.

We found that some EGFR-mutant cell sub-populations express specific markers (MetRes genes), which include laminin-binding, cytoskeletal re-modeling, and cell proliferation genes. These genes partially overlap with features of drug-resistant tumor cells identified in human NSCLCs by scRNA-seq^24^. Owing to the risks associated with craniotomies and CNS biopsies, it is difficult to obtain and study brain tissue from humans with advanced EGFR-mutant NSCLC, especially in those patients with CNS disease that developed after metastases appeared elsewhere in the body. Nevertheless, our pre-clinical and human correlates suggest that EGFR-mutant cell sub-populations with high competence for brain metastasis and TKI resistance may be detected in pre-treatment cancers or tumors first growing outside of the CNS. Therefore, it will be of significant interest to explore if MetRes genes can be used as dual markers of TKI resistance and CNS relapse, in the settings of early or advanced disease.

We discovered that RhoA and SRF enhance the outgrowth of persistent DTCs on laminin despite osimertinib treatment. RhoA and SRF also control the expression of several drug resistance and metastasis-associated genes in EGFR-mutant lung cancer cells. Some of these genes (e.g., *FOSL1*) were confirmed to be direct genomic targets of SRF in contractile fibroblasts^42^. Additional stromal factors may regulate the expression of these genes in the CNS. Alternatively, the apparent upregulation of these genes in vivo may be due to long-term selection of high MetRes expressing cell sub-populations in the brain. Ultimately, both tumor cell-intrinsic and extrinsic signals may shape the acquisition of MetRes features in brain metastatic cancer cells via RhoA and SRF. For instance, pre-existing cell populations that can express higher levels of SRF may be primed for subsequent selection and acquired drug resistance in response to the brain perivascular ECM. RhoA signaling is commonly thought to promote cell migration, particularly in neurons^48^. However, RhoA also regulates mechanosensing in response to internal cytoskeletal contractility or adhesion to the ECM^38^. During embryogenesis, subcellular RhoA activity is necessary to maintain epithelial cell polarity, by stabilizing integrin binding to BM laminin^39^. Consistent with this role of RhoA in epithelial cells, EGFR-mutant brain metastatic cells did not undergo EMT during adaptive phases of osimertinib treatment in vivo. It remains possible that transitions in cell state could correlate with invasion into other areas of the brain, or in some EGFR-mutant NSCLC cells that have undergone EMT prior to colonization in the CNS.

Finally, inhibition of RhoA had a particularly significant impact on EGFR-mutant NSCLC osimertinib resistance in the brain. In melanoma, RhoA can also promote drug resistance^49^, but the requirement for this pathway in CNS metastatic relapse had not been tested. It is possible that RhoA may contribute to multi-organ metastasis in the absence of drug. However, we evaluated the specific function of RhoA after DTCs seed the brain and respond to drug treatment. In this clinically relevant context, our data reveals a requirement for RhoA in enhancing brain metastatic progression following osimertinib treatment. We hypothesize that similar biological principals as well as various modifiers of RhoA activity will be involved in different physiological sites of drug persistent and resistant metastases. Characterizing distinct niches of residual disease in vivo and identifying subsets of MetRes genes, which regulate brain vs. multi-organ relapse to osimertinib or other TKIs, will be of interest for future therapeutic development.

## Methods

Our research complies with all relevant ethical regulations, including polices outlined by the Yale Institutional Animal Care, Yale Environmental Health and Safety committee, and Yale University Institutional Review Board for human research.

### Cell culture

The human cell population PC9-BrM4 was generated through four rounds of in vivo selection following intracardiac injection and brain colonization^18^. Prior to metastatic selection, the parental PC9 cells were infected with a lentivirus encoding a thymidine kinase, GFP, and luciferase reporter gene^50^. The C2 cell population was generated by performing an additional two rounds of in vivo selection following intracardiac injection and brain colonization of the PC9-BrM4 population. The R2 cell population was generated by performing an additional two rounds of in vivo selection for metastasis and resistance (see details below). H1975 were infected with a lentivirus reporter as mentioned above.

All cells were routinely tested for mycoplasma using the Universal mycoplasma detection kit (#30-1012k). All original ATCC cell stocks were authenticated using STAR PCR. Unless otherwise noted, cells were cultured as recommended by the American Type Culture Collection and in RPMI 1640 (Thermo Fisher Scientific #11875093) containing 10% FBS (Thermo Fisher Scientific #10437-028), 1% penicillin–streptomycin (Thermo Fisher Scientific #15140122), and 0.2% amphotericin B (Sigma Aldrich #A2942). All in vitro osimertinib treatments were done at a concentration of 160 nM unless otherwise noted.

### Animal experiments

All work was done in accordance with Yale Institutional Animal Care policies (protocol #201611338). Athymic mice (strain code:553 from NCI, male for PC9 derivatives, female for H1975, 5–6 weeks old) were purchased from Charles River Laboratories. For PDX engraftment, NSG female 5-week old mice were purchased from Jackson Laboratories (stock: 005557). All animals were acclimated for 7–10 days after arrival before experiments were initiated with at least weekly monitoring performed. Mice were housed in sex-matched groups (*n* = 5 mice per cage). Housing was standard micro-isolator caging; bedding material was 1/8th inch corn cob bedding, enriched for nest bedding. Husbandry conditions were light/dark cycle 12 h on, 12 h off at 72F ± 2F, and mice were fed with Envigo Teklad 2018S 18% protein rodent diet (sterilized). Access to food and water was *ad libitum*, and environmental enrichment was included (one square of cotton nesting material). Following the injection of tumor cells, animals were monitored daily for any signs of distress or poor body condition score. “Vehicle” refers to 0.5% methylcellulose. Osimertinib was reconstituted in 0.5% methylcellulose at a concentration of 6.25 mg/mL. Unless otherwise indicated, treatments (25 mg/mL in 100 μL) were administered five days per week via oral gavage.

### Tumor cell injection and monitoring

For intracardiac injections, 5e4 cells were resuspended in 100 μL sterile PBS and injected into the left ventricle. For intracranial injections, 5e3 cells or 2.8e4 cells (YU-006 PDX) were resuspended in 1 μL FBS and injected directly into the cerebrum of 8-week old NSG, using a digital mouse stereotactic instrument (Braintree Scientific; 51725-D). The injection site was 2 mm lateral from the bregma. Tumor growth was monitored by bioluminescent imaging using an IVIS Spectrum (Perkin-Elmer). 100 μL of 15 mg/mL d-luciferin (Perkin-Elmer #122796) dissolved in sterile PBS was injected retro-orbitally prior to imaging. Mice were imaged dorsally and ventrally, and the sum of the resulting signals was plotted. For ex vivo imaging, mice injected with d-luciferin were sacrificed, and the brains were immediately removed for imaging. Metastasis incidence was based on the detection of tumor derived luminescence at a given location (cranial or extracranial) that persisted through at least two subsequent timepoints. For progression-free survival (PFS), we used criteria as previously described for pre-clinical models^51^, where PFS is defined as time until tumor burden of treated mice reaches ~200% of the last recorded pre-treatment tumor burden. For subcutaneous injections, 2.5e6 cells were resuspended in 100 μL PBS mixed with 50% growth factor reduced Matrigel (Fisher Scientific; 356231) and injected into the hind flank. Tumor volume was measured with calipers and calculated using the formula (tumor volume = (major axis)(minor axis^2^) × 0.52). The maximal flank tumor size permitted by our ethics committee or institutional review board (1000 cubic mm) was not exceeded. Humane endpoints for animal studies and euthanasia were based on body condition score <2 (segmentation of vertebral column evident, dorsal pelvic bones are readily palpable) or a 20% loss in body weight.

Brains with osimertinib-resistant H1975 cranial lesions established via intracranial injection were collected following perfusion with 10 mL of PBS through the left ventricle. Following microdissection around the brain lesions, tissue was resuspended in 1.5 mg/mL collagenase III and 0.06 mg/mL Dnase1 in 1× HBSS with calcium and magnesium, homogenized by pulling up and down 2–3× with an 18-gauge needle and then a 21-gauge needle, and incubated at 37 °C for 10 min. The resulting solution was then centrifuged at 3446 × *g* for 3 min, resuspended in 5 mL PBS, passed through a 40 µM mesh filter, and spun down again. The resulting pellet was resuspended in 15 mL of 25% percoll (Fisher Scientific; MP219536980), prepared from an isotonic stock of 90% percoll and 10% 10× PBS, and spun at 4351 × g for 15 min with the accelerator and brake set to 1. The cell pellet was washed with 1× HBSS before being resuspended in PBS. GFP-positive H1975 tumor cells were sorted via Fluorescence assisted cell sorting (FACS) using an LSRII TAC6-GS instrument and Flow Jo v10.5. The gating strategy that was used is provided in Source Data 1c. Cells were immediately injected either subcutaneously or intracranially. Following confirmation of tumor cell engraftment (with bioluminescent imaging for intracranial injections and palpable tumors for subcutaneous injections), osimertinib treatment was initiated.

A patient with advanced lung cancer who developed acquired resistance to approved targeted agents provided informed written consent and enrolled to the Yale University Institutional Review Board–approved protocol (#1110009228), in accordance with ethical guidelines, allowing the collection and analysis of clinical data, archival, fresh tissue, and the generation of PDXs. YU-006 was obtained from a CT-guided biopsy of a white non-hispanic female patient that received Erlotinib treatment prior and Afatinib at the time of collection. Lung biopsy was implanted and maintained as subcutaneous PDX in vivo. The driver mutation EGFR E746_A750 [2235_2249del15]; T790M was validated by Sanger sequencing in the YU-006 PDX in accordance with patient biopsy. Prior to intracranial injection, YU-006 PDX was infected with a lentivirus encoding for zsGreen Luciferase (pHIV-Luc-ZsGreen was a gift from Bryan Welm (Addgene plasmid # 39196)).

### C2 and R2 model

Mice injected intracardially with PC9-BrM4 began treatment with vehicle or osimertinib when the whole-body tumor burden was 10× greater than the tumor burden on Day 0 of injection (23–44 days after injection). C1 and R1 populations were generated from moribund mice treated with either vehicle (14 days after the start of treatment) or osimertinib (112 days after the start of treatment), respectively, by macrodissecting brain lesions and collecting in 2% penicillin–streptomycin and 0.04% amphotericin B in PBS. Tissues were minced in 2% penicillin–streptomycin, 0.04% amphotericin B, 0.125% collagenase III, and 0.1% hyaluronidase in RPMI media for 1 h at 37 °C. Vortexing was performed every 15 min. Samples were then spun down, resuspended in 2% penicillin–streptomycin, 0.04% amphotericin B solution in 0.25 Trypsin, and incubated at 37 °C for 20 min. Two washes with 2% penicillin–streptomycin and 0.04% amphotericin B in PBS were performed before plating in standard tissue culture conditions in a T25 flask. 5e4 C1 and R1 cells in 100 μL sterile PBS were then reinjected into arterial circulation, and treatment and collection were reperformed as above, yielding the C2 and R2 cell populations. C2 and R2 cells were then infected with pLV-eGFP virus^52^. To confirm the selection of a resistant population, C2 and R2 cells were injected intracardially. Treatments were initiated 36 or 29 days after injection of the C2s and R2s, respectively.

### Desorption electrospray ionization mass spectrometry imaging (DESI-MSI)

Brains were collected 2 h after a single dose of osimertinib and at resistance for the R2 cells (defined as a tumor burden greater than 10x best response for the individual animal). All brains were embedded in axial position in a single block of 7.5w% HPMC ((hydroxypropyl)-methylcellulose, 40–60 cP) and 2.5w% PVP (polyvinylpyrrolidone, average mol wt. 360,000) hydrogel to ensure identical treatment of all brains during the following processing steps^53^. Tissue blocks were snap-frozen and subsequently tissue sections of 10 μm thickness were prepared using a Leica CM1950 cryostat. Sections were thaw mounted onto Superfrost glass slides, dried under a stream of nitrogen, and stored under −80 °C until analysis. Sections were collected approximately every 200 μm. Five slides were taken at each level of which one was stained using H&E for identifying the sections most suitable for MSI. H&E slides were digitalized using a Hamamatsu NanoZoomer 2.0 at 20× magnification (Hamamatsu, Japan). The slides chosen for MSI analysis showed the most and largest tumors.

DESI imaging experiments were performed using an automated 2D DESI source from Prosolia Inc. (Indianapolis, IN, USA) with a home-built sprayer assembly as described elsewhere^19^ mounted to a Thermo Q-Exactive instrument (Bremen, Germany). Analysis was performed in positive ion mode, with a mass range of *m/z* 200–900 and using 100 µm spatial resolution. Scan speeds were matched at 378.79 µm/s to achieve this lateral resolution at a mass resolution of 70,000 (AGC off, target 5E6) and injection time of 150 ms. S-Lens RF values of 75 were used and capillary temperature of 320 °C. Sprayer to sample distance was ~1.5 mm, sprayer to MS inlet distance was 7 mm, and inlet to sample distance «1 mm. Spray to sample angle was 75° and collection angle was 10°. Methanol/water (95:5 *v*/*v*) was used as electrospray solvent at a flow rate of 1.5 μL/min and a spray voltage of 4.5 kV. Solvent was delivered using a Dionex Ultimate 3000 nLC pump. Nebulizing gas pressure used was 6.5 bar of Nitrogen N4.8 (BOC).

Raw data was converted into.mzmL using the MSConvert tool (Proteowizard v3.0.4043)^54^ and then converted into.imzML using imzML Converter v1.3^55^. Image generation and intensity export was performed using ScilsLab 2018b (Bremen, Germany). Data were normalized using root-mean-square normalization and metastatic regions were defined in the MSI data by overlaying the H&E image and also using peaks localized specifically to the metastases. Osimertinib and metabolite intensities were exported from each metastatic area for each brain and the average intensity was then calculated for all metastatic areas combined. The values for each biological replicate were used for relative quantification performed using Prism (Graphpad) v7.1-v9.3.1. Details of statistical analyses are shown in figure legends.

### scRNA-sequencing

Three independent passages of PC9, PC9-BrM4, C2, and R2 cells were grown in culture and collected. The individual passages from each cell population were combined, and 10,000 viable cells (as determined by a Countess II Cell Counter (Life Technologies)) from each sample were submitted for sequencing. Nano-sized droplets containing a single cell with the bar-coded gel bead were generated using the Chromium controller (10× Genomics). Libraries were then created with Single Cell 3′ Library Kit V3 according to the manufacturer’s protocol. Reverse transcription was performed with polyT primers containing cell-specific bar codes, unique molecular identifiers, and adaptor sequences. 10x libraries were sequenced in an Illumina HISeq 4000 instrument. 10x Genomics Cell Ranger software v2.0.0 was used to align to the hg38 and its corresponding gene annotation, de-duplicate, filter bar codes, and quantify genes. For graph-based clustering and differential gene expression analysis, Seurat 3.0 workflow was used^56^. In Seurat, an initial filter was applied to select only the cells that had a minimum of 200 unique transcripts and to select only those genes that were expressed in at least three cells. For normalization and variance stabilization, the R package sctransform, which has a direct interface to Seurat toolkit, was employed^57^. During normalization with sctransform, mitochondrial mapping percentage was included in the model as an unwanted source of variation. Dimensionality reduction and graph-based clustering was performed on the transformed data with PCA and UMAP algorithm^57^.

Single-cell RNA-seq (scRNA-seq) data from human lung adenocarcinoma patients^24^ was obtained from https://github.com/czbiohub/scell_lung_adenocarcinoma and processed using scripts “NI08_Gene_expression_plotting.Rmd” and “NI16_cancercell_EGFR_ALK.Rmd**”**. Cells were subdivided into treatment-naïve, residual disease, and progressive disease and subsequently by disease driving mutation based on the annotations provided in the metadata. MetRes gene signature was calculated by differential mean, while single genes were plotted individually, based on the method used in the original script. Processed data was exported from R and analyzed using GraphPad Prism.

### BMX-seq analysis

Cerebrums were collected from the C2s and R2s after three days of either vehicle or osimertinib treatment (“early”). Samples for the “late” timepoint were collected from the R2s at resistance, when the tumor burden was greater than 10x best response for the individual animal, and time-matched C2s. All samples were collected and flash frozen 6 h after the last treatment. Samples harvested from in vitro cultures growing as a monolayer were harvested 24 h after treatment with vehicle or 160 nM osimertinib. Cell lysates were homogenized in QIAzol Lysis Reagent (QIAGEN; 79306) using an 18-gauge needle (in vivo samples) or a cell scraper (in vitro samples) (Corning; 353085) before being spun down in QIAshredder tubes (QIAGEN; 79656). RNA from in vivo and in vitro samples was extracted in parallel using the RNeasy Lipid Tissue mini kit (QIAGEN; 74804). Samples were sequenced on a HiSeq 4000 (Illumina) with paired-end 75 base pair reads.

Reads were trimmed of adapter sequences and then mapped to the combined gene annotation (GENCODE v24 for human, vM10 for mouse) using STAR. Differential gene expression was calculated by counting the uniquely mapped reads (MAPQ > = 10) to gene annotations using featureCounts and generating a count matrix of gene by sample^58^. This count matrix was then used as input to the DESeq2 R package^59^. The cross-mapping rate for a specific gene from all reads mapped to the opposite genome (mouse or human) was determined to exclude mouse reads inaccurately mapping to the human genome and vice versa. Mouse only (sham-injected animals from the lab’s previous BMX-seq datasets) and human only samples (C2 and R2 cells grown in vitro) were used as normalizing controls to estimate the cross-mapping rate to a human or mouse gene. Genes were excluded from analysis if the predicted cross-mapping reads of a gene exceeded 10% of the total reads mapped to that gene.

### Differential gene expression analysis

Enrichment analysis was conducted on gene lists filtered by significance (*P*-value < 0.05) using Metacore (Clarivate Analytics; v20.1.70000). The enrichment scores (calculated as –log10(*P*-value)) of those pathways and processes with the most significant *P*-values are plotted. GSEA 4.1 was performed using rank gene lists unfiltered for significance^60^. Rank gene lists were generated based on the direction of fold change multiplied by the inverse adjusted *P*-value of the comparison in question for each annotated transcript. The enrichment statistic used was “classic”. We considered genes to be differentially expressed when the *P*-value was <0.05 for the given comparison. PCA plots, hierarchical clustering (Pearson’s Dissimilarity), and intensity maps were generated in R package sctransform v0.2.0 and Partek Genomics Suite (Partek) PGS7.19.1125. MetRes signature score for all TCGA LUADs (*n* = 489 tumors)^61^ was calculated by averaging the median centered Variance Stabilization Transformation (VST) values for each gene in Supplementary Data 2. LUAD were classified as being MetRes high or MetRes low based on if their signature score was above or below the median of the cohort.

### Whole-exome sequencing

C2 and R2 cells growing in culture were washed with PBS, trypsinized, and spun down to form a pellet. For the four R2 Late osimertinib-treated samples, FACS was used to collect GFP-positive tumor cells after brain digestion (same as above). Genomic DNA was then extracted (Qiagen; #51306). 1.0 µg of genomic DNA was sheared to a mean fragment length of about 140 base pairs using focused acoustic energy (Covaris E210). Exome sequencing was performed by exome capture using the IDT xGen capture probe panel with an additional “spike-in” of ~2500 regions, totaling ~620 kb, of RefGene coding regions that were not included or were poorly covered by the IDT panel. Captured fragments were sequenced using 101 base pair paired-end sequencing reads in an Illumina NovaSeq 6000 with a S4 flowcell according to Illumina protocols. Sequencing reads were aligned to human genome build 38 (GRCh38/hg38) using the BWA-MEM v0.7.15, aggregated into a BAM file, and further processed to produce variants with GATK v3.4, following the GATK Best Practices workflow^62–64^.

Variants were annotated with ANNOVAR 2017Jul16, and MetaSVM was used to predict the deleteriousness of non-synonymous variants^65,66^. For rare transmitted dominant variants, only LoF mutations (stop-gains, stop-losses, canonical splice-sites, and frameshift indels) and D-mis mutations (missense mutations predicted deleterious by MetaSVM) were considered potentially damaging and filtered using the following criteria to reduce false positives: (1) GATK variant quality score recalibration (VQSR) of PASS, (2) MAF ≤ 2 × 10–5 in gnomAD v2.1 (calculated based on combined dataset of WES and WGS data from gnomAD database)^67^, (3) DP ≥ 8 independent reads, (4) GQ score ≥ 20, (5) MQ score ≥ 40, (6) PLdiff/DP ≥ 8, and (7) Low Complexity Regions (LCRs) were also excluded. Transmitted recessive variants were filtered for rare (MAF ≤ 10 − 3 in gnomAD) homozygous and compound heterozygous variants using the same criteria described above. The list of variants was further filtered according to the following criteria (as adapted from^68^): more than 11 reads in Cv2D and Rv2D, more than two non-reference reads in Rv2D, more than two reference reads in Cv2D, non-reference allele frequency (NRAF) greater than 0.15 in Rv2D, difference between Rv2D and Cv2D NRAF greater than 0.25, and the presence of the variant in all four RoL samples. Next, we further filtered for variants in genes with an average RPKM greater than 10 across all samples. REVEL scores for the four missense mutations were determined according to^26^.

### Immunohistochemistry and immunofluorescence

Brains from C2 and R2 mice at all BMX-seq timepoints, from YU-006 mice and from H1975 injected mice were collected from euthanized mice. Tissues were fixed overnight in 4% PFA at 4 °C, rinsed with PBS, and embedded in paraffin for immunohistochemistry or in OCT after shaking at 4 °C in 30% sucrose for at least 48 h for immunofluorescence staining. Paraffin (5 µm) and frozen blocks (20 µm) were sectioned. For immunostaining, slides were blocked in 3% BSA/TBS 0.1% Tween and 0.5% Triton-X100 for 30 min at room temperature. Primary antibodies were incubated overnight at 4 °C in 0.3% BSA/TBST with DAPI (0.5 µg/mL). Secondary antibodies were incubated at room temperature for 1 h in 0.3% BSA/TBST, and slides were mounted in ProLong® Gold Antifade Reagent (Cell Signaling; #9071). For immunohistochemistry on paraffin slides, antigen retrieval was performed using EDTA (buffer pH = 8.0). Peroxidase activity was blocked using 3% H_2_O_2_ in PBS after antigen retrieval. Slides were incubated using Vector ABC reagent and developed using NovaRed Peroxidase (Vector Laboratories Inc), counterstained with Hematoxylin, and mounted in Permount mounting media (Electronic Microscopy Science; #17986-01). The primary antibodies used are: phospho-EGFR (Cell Signaling Technologies (CST), #3777), Collagen-IV (Millipore, AB756), Laminin 1/2 (Abcam, Ab7463), CD34 (Abcam, Ab8158), phospho-Histone H3 (pHH3) (CST, #9701), Cleaved Caspase-3 (CC3) (CST, #9661), Pan Cytokeratin (E-Bioscience, 53-9003-8), phospho-MLC2 (CST, #3674). The secondary antibodies used are from Jackson Immunoresearch: Alexa Fluor® 647 AffiniPure Donkey Anti-Rabbit IgG (H + L) (711-605-152), Rhodamine Red™-X (RRX) AffiniPure Donkey Anti-Rat IgG (H + L) (712-295-153), Alexa Fluor® 488 AffiniPure Donkey Anti-Mouse IgG (H + L) (715-545-151). See Supplementary Table 2 for antibody dilutions.

### Image processing

All images were obtained using a Keyence microscope (BZ-X700) at 4×, 10×, 20×, or 40× magnification. Images that are directly compared were stained at the same time, imaged with the same exposure time, and processed in parallel. All image processing, compiling, and quantification was performed using ImageJ software v1.52-v1.53. For the quantification of laminin adjacent tumor cells, GFP-positive tumor cells (threshold 0–40) that were directly adjacent to laminin staining (threshold 10–80) were manually counted. For the quantification of pHH3, a macro was generated in which ROIs were generated using the DAPI images (threshold 0–12). It was then determined whether each ROI was GFP-positive (indicating tumor cells) (threshold 0–8) and whether each ROI was pHH3+ (threshold 0–25). The % of GFP+ nuclei that are pHH3+ was plotted. Quantification of laminin intensity was done using ImageJ. ROIs were defined for each GFP + tumor lesion/cluster and applied to laminin images to measure mean intensity.

### Cellular growth assays

For IC_50_ analysis, 2.5e3 cells for PC9 derivatives were seeded in traditional tissue culture 96-well plates. 24 h later, the media was replaced with media containing increasing concentrations of osimertinib in triplicate. Seventy-two hours later, cell viability was measured using resazurin (RD Systems #AR002). At the time of analysis, media was removed, and 100 μL of diluted resazurin (1:10 in PBS) was added to each well. Plates were incubated for 30 min at 37 °C and 5% CO_2_. The resulting fluorescence was read at 560/610 nm (ex/em) using a Synergy MX Multi-Mode Microplate Reader (BioTek).

For colony outgrowth assays*,* 500 cells were seeded in triplicate in standard tissue culture 24-well plates (Corning; 3526) or 24-well plates coated with collagen I (Corning; 354408), collagen-IV (Corning; 354430), fibronectin (Corning; 354411), or laminin (Corning; 354412). Twenty-four hours later, media was replaced with media containing vehicle or 160 nM osimertinib. Media was refreshed every 3–4 days for 18–21 days. For shRNAs induction, Dox (0.5 μg/mL) was added to all treatment media. For experiments using sgRNAs, Dox (0.5 μg/mL) was added in the initial treatment media and was removed after 4 days of Dox treatment. At endpoint, treatment media was removed, and 400 μL of media containing 150 µg/mL D-luciferin (Perkin-Elmer #122796) was added to each well. Plates were incubated at 37 °C for 10 min and bioluminescent imaging was performed on an IVIS Spectrum. Cells were then rinsed with PBS and fixed in 4% PFA for 10 min at RT. PFA was removed and replaced with 0.05% crystal violet for 2 h. Plates were rinsed three times with water and left to dry before being imaged on a ChemiDoc Imaging System (Bio-Rad).

### Species-specific quantitative PCR

Tumor-bearing hemispheres were collected and processed as described above for BMX-seq. PCR of cDNA was completed using TaqMan Universal Master Mix no AmpErase UNG (Applied Biosystems; 4324018). Species-specific TaqMan primers (20x) were designed and ordered through Applied Biosystems. qRT-PCR reactions were run in triplicate, data was normalized to housekeeping gene *HPRT1* (human), and relative quantification (RQ) values were calculated via the 2^–ΔΔCt^ method. All qPCRs from in vivo samples are shown as the mean fold change across indicated samples ± SEM. All qPCRs from in vitro samples are shown as the mean fold change across indicated samples ± SD. All TaqMan probes were purchased from Applied Biosystems/Thermo Fisher Scientific. See Supplementary Table 3 for qPCR probes assay IDs.

### Western blotting

Cells were rinsed with PBS and lysed directly in the plate using RIPA buffer with protease inhibitors (Roche #11836170001) and phosphatase inhibitors (Sigma #P5726 and #P0044). Cells were lysed on ice for 30 min and lysates were clarified by centrifugation. Protein was quantified and analyzed by SDS-PAGE using the Mini-PROTEAN electrophoresis system (Bio-Rad). Protein was transferred to nitrocellulose by wet transfer and membranes blocked using 5% milk in TBST (0.1% Tween20). Blots were incubated with primary antibodies at 4 °C overnight, then HRP-secondary antibodies for 1 h at room temperature. ECL was used to develop blots, which were imaged with a ChemiDoc Imaging System (Bio-Rad). For experiments on laminin-coated plates (Corning; 354404), cells were plated and allowed to grow for three days before being serum starved (0.2% FBS) overnight. Cells were then treated with 160 nM osimertinib in 10% FBS RPMI. The primary antibodies used are: ITGB1 (Cell Signaling Technologies (CST), #9699), Tubulin (SIGMA, T5168), GAPDH (CST, #2118), phospho-Cofilin (CST, #3313), Cofilin (CST, #5175), phospho-EGFR (CST, #3777), EGFR (CST, #2232), phospho-AKT (CST, #4056), AKT (CST, #4691), phospho-ERK (CST, #4370), SRF (CST, #5147), phospho-YAP (Abcam, #76252), YAP (CST, #8418), MKL-1 (Bethyl, #A302-201A-M). The secondary antibodies used are: Anti-mouse Horse Radish Peroxidase (HRP) (Thermo Scientific, #31437) and anti-rabbit HRP (Thermo Scientific, #31458). See Supplementary Table 2 for antibody dilutions.

### Gene knockdown

sgRNAs were subcloned into a lentiCRISPRv2 one vector system^69^. sgRNA targeting Rosa26 was used as a control (sgCntrl). Knockdown was confirmed with Western blotting after 4–5 days of treatment with Dox (0.5 µg/mL) in samples lysed immediately or after remaining in culture for 13 days without Dox. shRNAs were subcloned into the pINDUCER10 vector using the Mlu1 and Xho1 restriction sites. Viruses were produced using Mirus Bio transfection reagent and 293T cells following the manufacturer’s instructions. shRNA targeting Arab2 was used as a control (shCntrl). Knockdown was confirmed with qRT-PCR after 4–5 days of treatment with Dox (0.5 µg/mL) in samples lysed immediately. See Supplementary Table 4 for sgRNA and shRNA target sequence.

### Statistical analysis

Data is presented as mean ± SEM (unless otherwise noted) with *P*-values calculated by two-tailed Student’s *t*-test, Welch’s *t*-test, Mann–Whitney, or ANOVA depending on whether the dataset has a normal distribution and whether there is significant variance. In all cases, *P*-value < 0.05 is considered significant. For all mouse experiments showing tumor burden over time, *P*-value is calculated based on area under the curve (AUC). *P*-values for metastasis-free survival or progression-free survival were calculated by log-rank test. All statistics were calculated using Prism (Graphpad) v7.1-v9.3.1) or Partek Genomics Suite (Partek) PGS7.19.1125.

## Supplementary information

Supplementary Information

Supplementary Data 1

Supplementary Data 2

Supplementary Data 3

Description of Additional Supplementary Files

Soure data file

## Data Availability

The publicly available Human genome build 38 (GRCh38/hg38) is deposited in GenBank assembly under the accession number GCA_000001405.29. The publicly available GENCODE v24 for human is deposited at GENCODE and available at https://www.gencodegenes.org/human/release_24.html. The publicly available vM10 for mouse is deposited at GENCODE and available at https://www.gencodegenes.org/mouse/release_M10.html. The publicly available single-cell RNA-seq from LUAD patients used in this study was deposited as an NCBI BioProject #PRJNA591860^24^ and available at https://github.com/czbiohub/scell_lung_adenocarcinoma. RNA-seq and WES data generated for this study are deposited in GEO under the accession codes GSE174850, GSE174851, and GSE174852. The remaining data are provided in the Article file, Supplementary Information, Supplementary Data, and Source Data file.

## References

[1] Cagney DN (2017). Incidence and prognosis of patients with brain metastases at diagnosis of systemic malignancy: a population-based study. Neuro Oncol..

[2] Stelzer KJ (2013). Epidemiology and prognosis of brain metastases. Surg. Neurol. Int.

[3] Shin DY (2014). EGFR mutation and brain metastasis in pulmonary adenocarcinomas. J. Thorac. Oncol..

[4] Rangachari D (2015). Brain metastases in patients with EGFR-mutated or ALK-rearranged non-small-cell lung ca*ncers*. Lung Cancer.

[5] Ouyang W (2020). Metachronous brain metastasis in patients with EGFR-mutant NSCLC indicates a worse prognosis. J. Cancer.

[6] Politi K, Ayeni D, Lynch T (2015). The next wave of EGFR tyrosine kinase inhibitors enter the clinic. Cancer Cell.

[7] Park SJ (2012). Efficacy of epidermal growth factor receptor tyrosine kinase inhibitors for brain metastasis in non-small cell lung cancer patients harboring either exon 19 or 21 mutation. Lung Cancer.

[8] Omuro AM (2005). High incidence of disease recurrence in the brain and leptomeninges in patients with nonsmall cell lung carcinoma after response to gefitinib. Cancer.

[9] Lee YJ (2010). Frequent central nervous system failure after clinical benefit with epidermal growth factor receptor tyrosine kinase inhibitors in Korean patients with nonsmall-cell lung cancer. Cancer.

[10] Lee JS (2019). The impact of systemic treatment on brain metastasis in patients with non-small-cell lung cancer: A retrospective nationwide population-based cohort study. Sci. Rep..

[11] Ballard P (2016). Preclinical comparison of osimertinib with other EGFR-TKIs in EGFR-mutant NSCLC brain metastases models, and early evidence of clinical brain metastases activity. Clin. Cancer Res..

[12] Colclough N (2021). Preclinical comparison of the blood-brain barrier permeability of osimertinib with other EGFR TKIs. Clin. Cancer Res..

[13] Wu YL (2020). Osimertinib in resected EGFR-mutated non-small-cell lung cancer. N. Engl. J. Med..

[14] Wu YL (2018). CNS efficacy of osimertinib in patients with t790m-positive advanced non-small-cell lung cancer: data from a randomized phase III trial (AURA3). J. Clin. Oncol..

[15] Reungwetwattana, T. et al. CNS response to osimertinib versus standard epidermal growth factor receptor tyrosine kinase inhibitors in patients with untreated EGFR-mutated advanced non-small-cell lung cancer. *J. Clin. Oncol.* Jco2018783118, 10.1200/jco.2018.78.3118 (2018). 10.1200/JCO.2018.78.3118 30153097

[16] Cross DA (2014). AZD9291, an irreversible EGFR TKI, overcomes T790M-mediated resistance to EGFR inhibitors in lung cancer. Cancer Discov..

[17] Guo S, Jiang X, Mao B, Li QX (2019). The design, analysis and application of mouse clinical trials in oncology drug development. BMC Cancer.

[18] Nguyen DX (2009). WNT/TCF signaling through LEF1 and HOXB9 mediates lung adenocarcinoma metastasis. Cell.

[19] Takáts Z, Wiseman JM, Gologan B, Cooks RG (2004). Mass spectrometry sampling under ambient conditions with desorption electrospray ionization. Science.

[20] Hall A (2012). Rho family GTPases. Biochem. Soc. Trans..

[21] Kubala MH, DeClerck YA (2019). The plasminogen activator inhibitor-1 paradox in cancer: a mechanistic understanding. Cancer Metastasis Rev..

[22] Vallejo A (2017). An integrative approach unveils FOSL1 as an oncogene vulnerability in KRAS-driven lung and pancreatic cancer. Nat. Commun..

[23] Wulf MA, Senatore A, Aguzzi A (2017). The biological function of the cellular prion protein: an update. BMC Biol..

[24] Maynard A (2020). Therapy-induced evolution of human lung cancer revealed by single-cell RNA sequencing. Cell.

[25] Wingrove E (2019). Transcriptomic hallmarks of tumor plasticity and stromal interactions in brain metastasis. Cell Rep..

[26] Ioannidis NM (2016). REVEL: an ensemble method for predicting the pathogenicity of rare missense variants. Am. J. Hum. Genet.

[27] Karczewski KJ (2020). The mutational constraint spectrum quantified from variation in 141,456 humans. Nature.

[28] Sayyah J (2014). The Ras-related protein, Rap1A, mediates thrombin-stimulated, integrin-dependent glioblastoma cell proliferation and tumor growth. J. Biol. Chem..

[29] Waseem A (1998). Isolation, sequence and expression of the gene encoding human keratin 13. Gene.

[30] Kienast Y (2010). Real-time imaging reveals the single steps of brain metastasis formation. Nat. Med..

[31] Thomsen MS, Routhe LJ, Moos T (2017). The vascular basement membrane in the healthy and pathological brain. J. Cereb. Blood Flow. Metab..

[32] Kurihara M (2019). Rapidly progressive miliary brain metastasis of lung cancer after EGFR tyrosine kinase inhibitor discontinuation: An autopsy report. Neuropathology.

[33] Iguchi Y (2007). Miliary brain metastases from adenocarcinoma of the lung: MR imaging findings with clinical and post-mortem histopathologic correlation. Neuroradiology.

[34] Hsu F, Nichol A, Toriumi T, De Caluwe A (2017). Miliary metastases are associated with epidermal growth factor receptor mutations in non-small cell lung cancer: a population-based study. Acta Oncol..

[35] Carbonell WS, Ansorge O, Sibson N, Muschel R (2009). The vascular basement membrane as “soil” in brain metastasis. PLoS ONE.

[36] Albrengues, J. et al. Neutrophil extracellular traps produced during inflammation awaken dormant cancer cells in mice. *Science* **361**, 10.1126/science.aao4227 (2018). 10.1126/science.aao4227 PMC6777850 30262472

[37] Ramovs V, Te Molder L, Sonnenberg A (2017). The opposing roles of laminin-binding integrins in cancer. Matrix Biol..

[38] Huveneers S, Danen EH (2009). Adhesion signaling-crosstalk between integrins, Src and Rho. J. Cell Sci..

[39] Nakaya Y, Sukowati EW, Wu Y, Sheng G (2008). RhoA and microtubule dynamics control cell-basement membrane interaction in EMT during gastrulation. Nat. Cell Biol..

[40] Kim JG (2018). Regulation of RhoA GTPase and various transcription factors in the RhoA pathway. J. Cell Physiol..

[41] Chen R, Xie R, Meng Z, Ma S, Guan KL (2019). STRIPAK integrates upstream signals to initiate the Hippo kinase cascade. Nat. Cell Biol..

[42] Foster CT, Gualdrini F, Treisman R (2017). Mutual dependence of the MRTF-SRF and YAP-TEAD pathways in cancer-associated fibroblasts is indirect and mediated by cytoskeletal dynamics. Genes Dev..

[43] Igawa S (2019). Impact of EGFR genotype on the efficacy of osimertinib in EGFR tyrosine kinase inhibitor-resistant patients with non-small cell lung cancer: a prospective observational study. Cancer Manag. Res..

[44] Zheng Q (2020). EGFR mutation genotypes affect efficacy and resistance mechanisms of osimertinib in T790M-positive NSCLC patients. Transl. Lung Cancer Res..

[45] Foggetti G (2021). Genetic determinants of EGFR-driven lung cancer growth and therapeutic response in vivo. Cancer Discov..

[46] Biswas AK (2022). Targeting S100A9-ALDH1A1-retinoic acid signaling to suppress brain relapse in EGFR-mutant. Lung Cancer Cancer Discov..

[47] Quail DF, Joyce JA (2017). The microenvironmental landscape of brain tumors. Cancer Cell.

[48] Hall A, Lalli G (2010). Rho and Ras GTPases in axon growth, guidance, and branching. Cold Spring Harb. Perspect. Biol..

[49] Misek SA (2020). Rho-mediated signaling promotes BRAF inhibitor resistance in de-differentiated melanoma cells. Oncogene.

[50] Ponomarev V (2004). A novel triple-modality reporter gene for whole-body fluorescent, bioluminescent, and nuclear noninvasive imaging. Eur. J. Nucl. Med. Mol. Imaging.

[51] Gao H (2015). High-throughput screening using patient-derived tumor xenografts to predict clinical trial drug response. Nat. Med..

[52] Enomoto M, Bunge MB, Tsoulfas P (2013). A multifunctional neurotrophin with reduced affinity to p75NTR enhances transplanted Schwann cell survival and axon growth after spinal cord injury. Exp. Neurol..

[53] Dannhorn A (2020). Universal sample preparation unlocking multimodal molecular tissue imaging. Anal. Chem..

[54] Chambers MC (2012). A cross-platform toolkit for mass spectrometry and proteomics. Nat. Biotechnol..

[55] Race AM, Styles IB, Bunch J (2012). Inclusive sharing of mass spectrometry imaging data requires a converter for all. J. Proteom..

[56] Butler A, Hoffman P, Smibert P, Papalexi E, Satija R (2018). Integrating single-cell transcriptomic data across different conditions, technologies, and species. Nat. Biotechnol..

[57] Becht E (2019). Dimensionality reduction for visualizing single-cell data using UMAP. Nat. Biotechnol..

[58] Liao Y, Smyth GK, Shi W (2014). featureCounts: an efficient general purpose program for assigning sequence reads to genomic features. Bioinformatics.

[59] Love MI, Huber W, Anders S (2014). Moderated estimation of fold change and dispersion for RNA-seq data with DESeq2. Genome Biol..

[60] Subramanian A (2005). Gene set enrichment analysis: a knowledge-based approach for interpreting genome-wide expression profiles. Proc. Natl Acad. Sci. USA.

[61] The Cancer Genome Atlas Research Network. Comprehensive molecular profiling of lung adenocarcinoma. *Nature* **511**, 543–550 (2014). 10.1038/nature13385 PMC4231481 25079552

[62] McKenna A (2010). The Genome Analysis Toolkit: a MapReduce framework for analyzing next-generation DNA sequencing data. Genome Res..

[63] Van der Auwera GA (2013). From FastQ data to high confidence variant calls: the Genome Analysis Toolkit best practices pipeline. Curr. Protoc. Bioinforma..

[64] Li, H. Aligning sequence reads, clone sequences and assembly contigs with BWA-MEM. https://arxiv.org/abs/1303.3997 (2013).

[65] Wang K, Li M, Hakonarson H (2010). ANNOVAR: functional annotation of genetic variants from high-throughput sequencing data. Nucleic Acids Res..

[66] Dong C (2014). Comparison and integration of deleteriousness prediction methods for nonsynonymous SNVs in whole exome sequencing studies. Hum. Mol. Genet..

[67] Lek M (2016). Analysis of protein-coding genetic variation in 60,706 humans. Nature.

[68] Jacob LS (2015). Metastatic competence can emerge with selection of preexisting oncogenic alleles without a need of new mutations. Cancer Res..

[69] Shalem O (2014). Genome-scale CRISPR-Cas9 knockout screening in human cells. Science.

