# Supplementary material for: Brain metastatic outgrowth and osimertinib resistance are potentiated by RhoA in EGFR-mutant lung cancer
Source: Nat Commun. 2022 Dec 12;13:7690. doi: 10.1038/s41467-022-34889-z (PMC9744876; doi:10.1038/s41467-022-34889-z)
Supplement: Supplementary file 1 — Supplementary Information [file 41467_2022_34889_MOESM1_ESM.pdf]

Supplementary Information

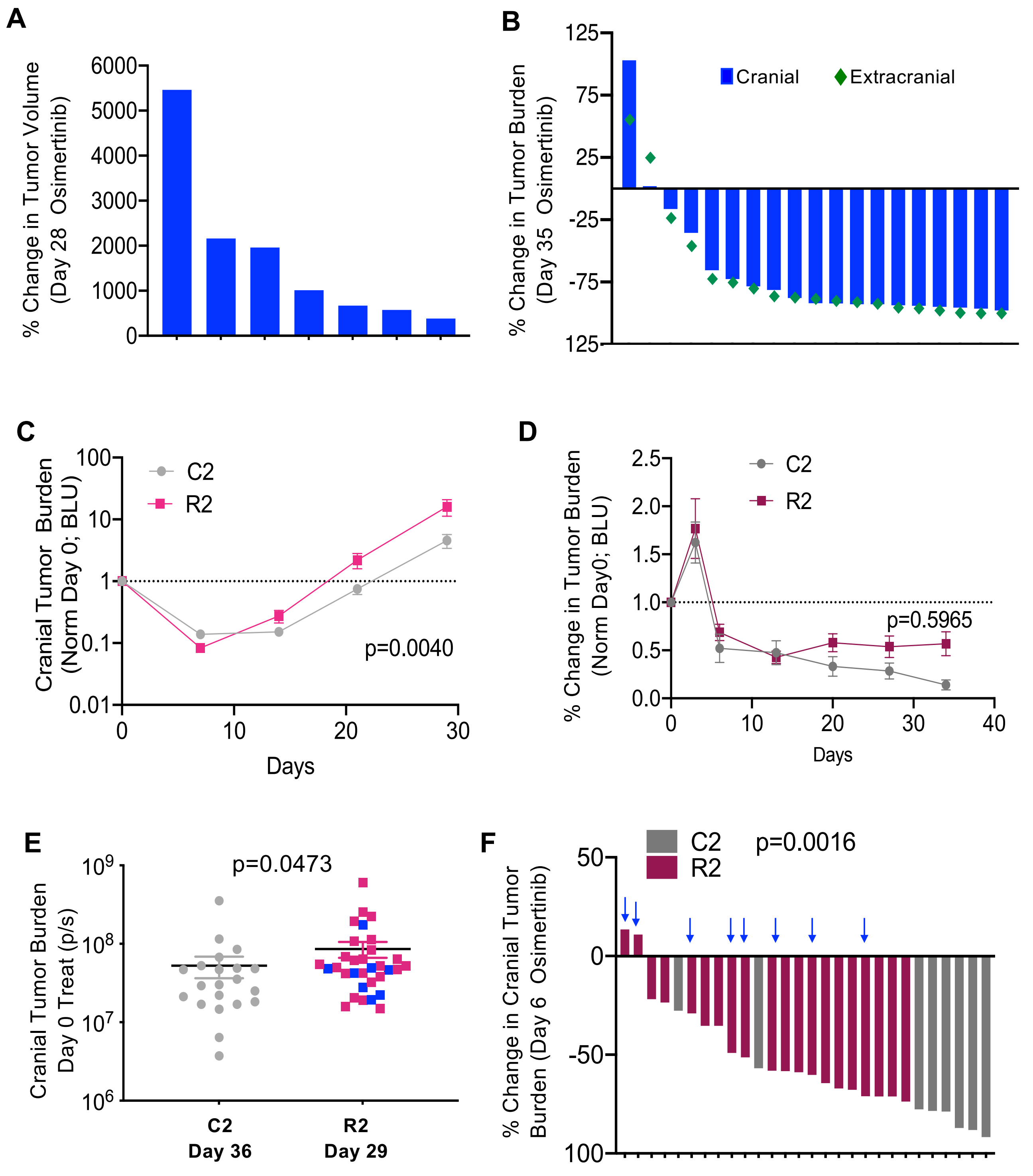

Supplementary Figure 1

Supplementary Figure 1 (Related to Figure 1)

(A) Waterfall plot depicting % change of H1975 cranial tumor burden from Figure 1A from Day 0 to Day 28 of treatment with osimertinib. **(B)** Waterfall plot depicting % change of PC9-BrM4 metastases from Figure 1D. Blue bar indicates % change in cranial BLI and green diamond indicates % change in extracranial BLI of the same animal from Day 0 to Day 35 of treatment with osimertinib. **(C)** Cranial tumor growth of mice injected with C2 and R2 cells prior to treatment with osimertinib. For each animal, tumor burden is determined by BLI at a given time point and is normalized to BLI at Day 0 of tumor cell injection. N=21 animals for C2, and N=32 animals for R2. Data presented as mean values +/- SEM. P-value calculated based on AUC by Mann-Whitney (two-sided). **(D)** Extracranial tumor burden of the same mice in Figure 1G. N=4 animals for C2, and N=15 animals for R2. Data presented as mean values +/- SEM. P-value calculated based on AUC by Mann-Whitney (two-sided). **(E)** Dot plot showing cranial tumor burden of C2 and R2 injected mice from Figure 1G, at Day 36 and Day 29 after intra-cardiac injection for C2 and R2 cells, respectively. Blue dots indicate the starting brain BLI of animals denoted by blue arrows in (F). N=21 animals for C2, and N=32 animals for R2. Data presented as mean values +/- SEM. P-value calculated by Mann-Whitney (two-sided). **(F)** Waterfall plot depicting % change in cranial bioluminescence from Day 0 to Day 6 of treatment in the same animals as in (E). % response of R2 cranial tumors to osimertinib indicated by blue arrows does not corelate with their starting tumor burden (indicated by blue dots in E). N=7 animals for C2, and N=21 animals for R2. P-value calculated by Mann-Whitney (two-sided).

A

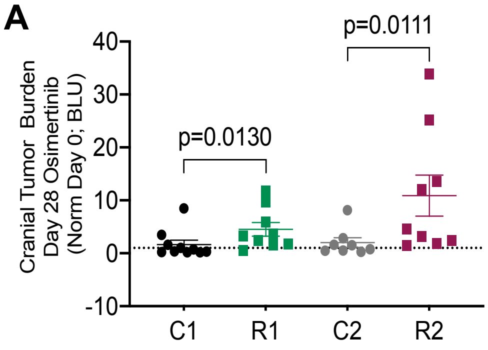

B

Injection Type	Day of Comparison	C2 (n=X)	R2 (n=X)	Fold Change Cranial Tumor Burden (R2 vs. C2)	p-value
Intracardiac 1	35	8	6	8.17	0.0080
Intracardiac 2	34	4	15	4.75	0.0186
Intracardiac 3	36	9	12	2.88	0.0148
Intracranial	38	5	6	88.60	0.0303

C

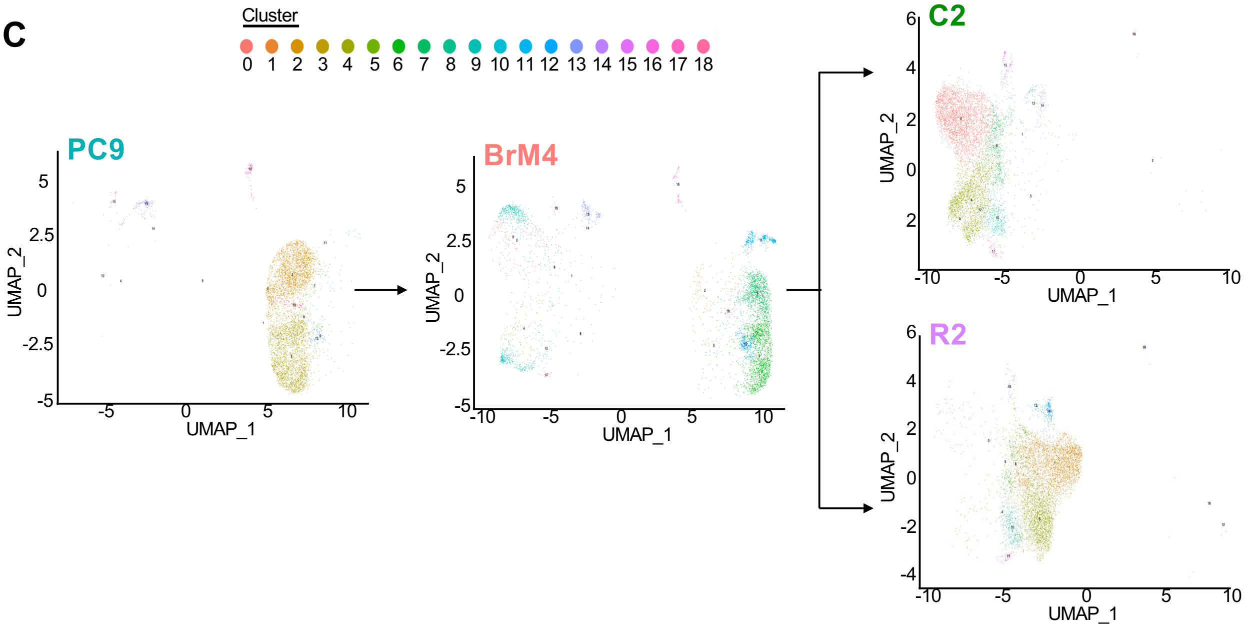

D

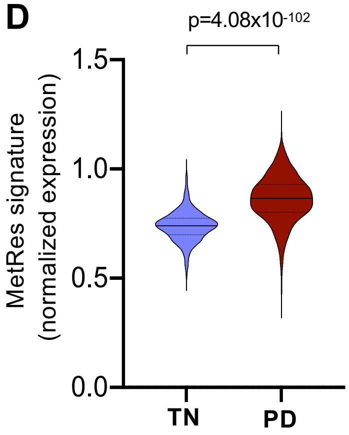

E

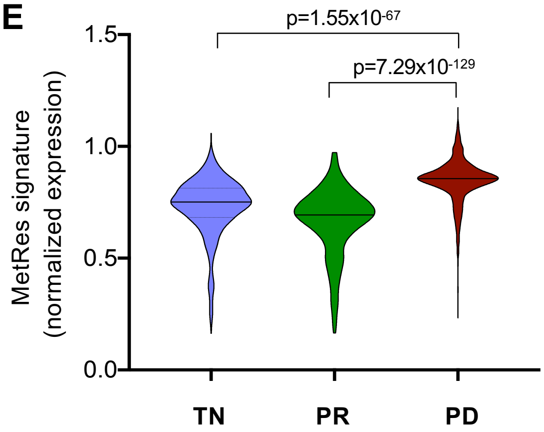

Supplementary Figure 2

Supplementary Figure 2 (Related to Figure 3)

(A) Dot plot indicating cranial tumor burden of mice with C1, R1, C2, and R2 brain metastases treated with osimertinib for 28 days. Tumor burden is measured by BLI and is normalized to Day 0 of treatment for each animal C1. (N= 10 animals), R1 (N=9 animals), C2 (N=8 animals), R2 (N=9 animals). Data presented as mean values +/- SEM. P-values calculated by Mann-Whitney (two-sided). **(B)** Table summarizing four independent experiments comparing the cranial tumor burden of C2 and R2 metastasis formed after intra-cardiac or intra-cranial injection of tumor cells and treated with osimertinib. X is the number of animals per group (N). P-value was calculated by Mann-Whitney (two-sided). **(C)** Uniform Manifold Approximation and Projection (UMAP) was re-plotted for PC9, PC9-BrM4, C2, or R2 bulk populations separately and annotated for cell sub-populations identified in Figure 3C. **(D)** Expression of the indicted genes was plotted as in Figure 3G, but for ALK mutant patients (N=6 patients). TN=treatment naïve patients (N=360 cells). PD=patients with progressive disease after systemic therapy (N=873 cells). **(E)** Expression of the indicted genes was plotted as in Figure 3G using all available samples (N=22 patients). TN=treatment naïve patients (N=1073 cells). RD=Patients with partial responses (N=572 cells). PD=patients with progressive disease (N=2109 cells). P-values for D and E calculated by Welch's t-test (two-sided).

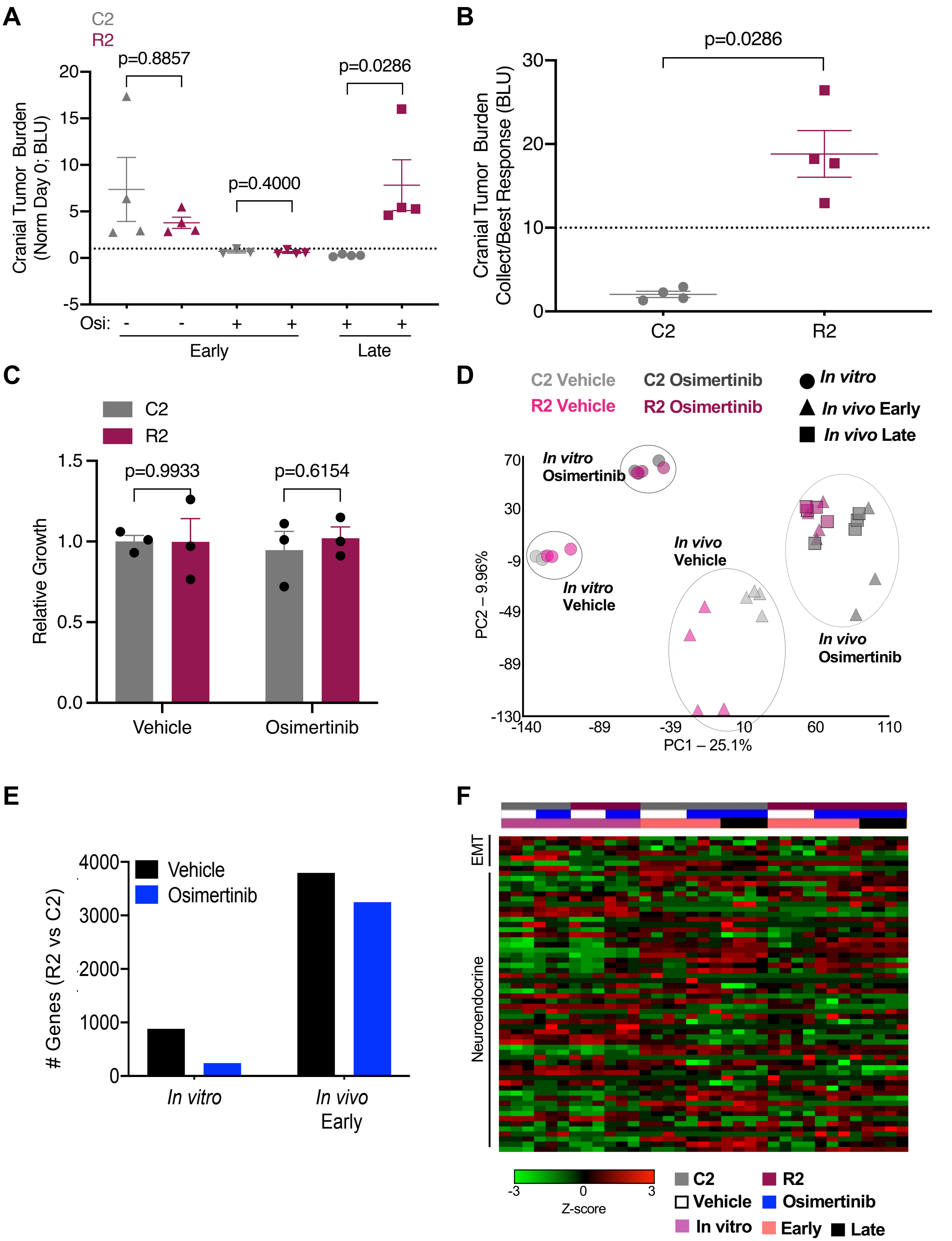

Supplementary Figure 3

Supplementary Figure 3 (Related to Figure 4)

(A) Dot plot showing cranial tumor cell burden of mice from Figure 4A at the time of brain tissue collection for BMX-seq. “Early” indicates three days of treatment and “late” indicates 34-58 days of treatment. All brains were collected six hours after the last dose for osimertinib. Metastatic tumor burden is determined by BLI and is normalized to Day 0 of treatment for each animal. N=4 animals, except for C2 early/Osimertinib N=3 animals. Data presented as mean +/- SEM. P-value calculated by Mann-Whitney (two-sided). **(B)** Dot plot showing cranial tumor burden of R2 metastases treated with osimertinib at the late timepoints from (A), but normalized to the corresponding animal’s best response to osimertinib. N=4 animals per group. Data presented as mean values +/- SEM. P-value was calculated by Mann-Whitney (two-sided). **(C)** Cell count from C2 and R2 cells in vitro treated with osimertinib for 24 hours. All values normalized to C2 vehicle treated samples. N=3 per group. Data presented as mean +/- SEM. P-value calculated by t-test (two-sided). **(D)** Principal component (PC) analysis using the gene expression of C2 and R2 tumor cells grown *in vitro* or in the brain *in vivo* and treated with either vehicle or osimertinib (160nM *in vitro* and 25mg/kg *in vivo*). **(E)** Plotted are the number of genes that are significantly differentially expressed in R2 tumor cells compared to C2 tumor cells *in vitro* or *in vivo*. **(F)** Heatmap depicting normalized expression of EMT and neuroendocrine lineage genes in tumor cells of the indicated brain metastasis samples.

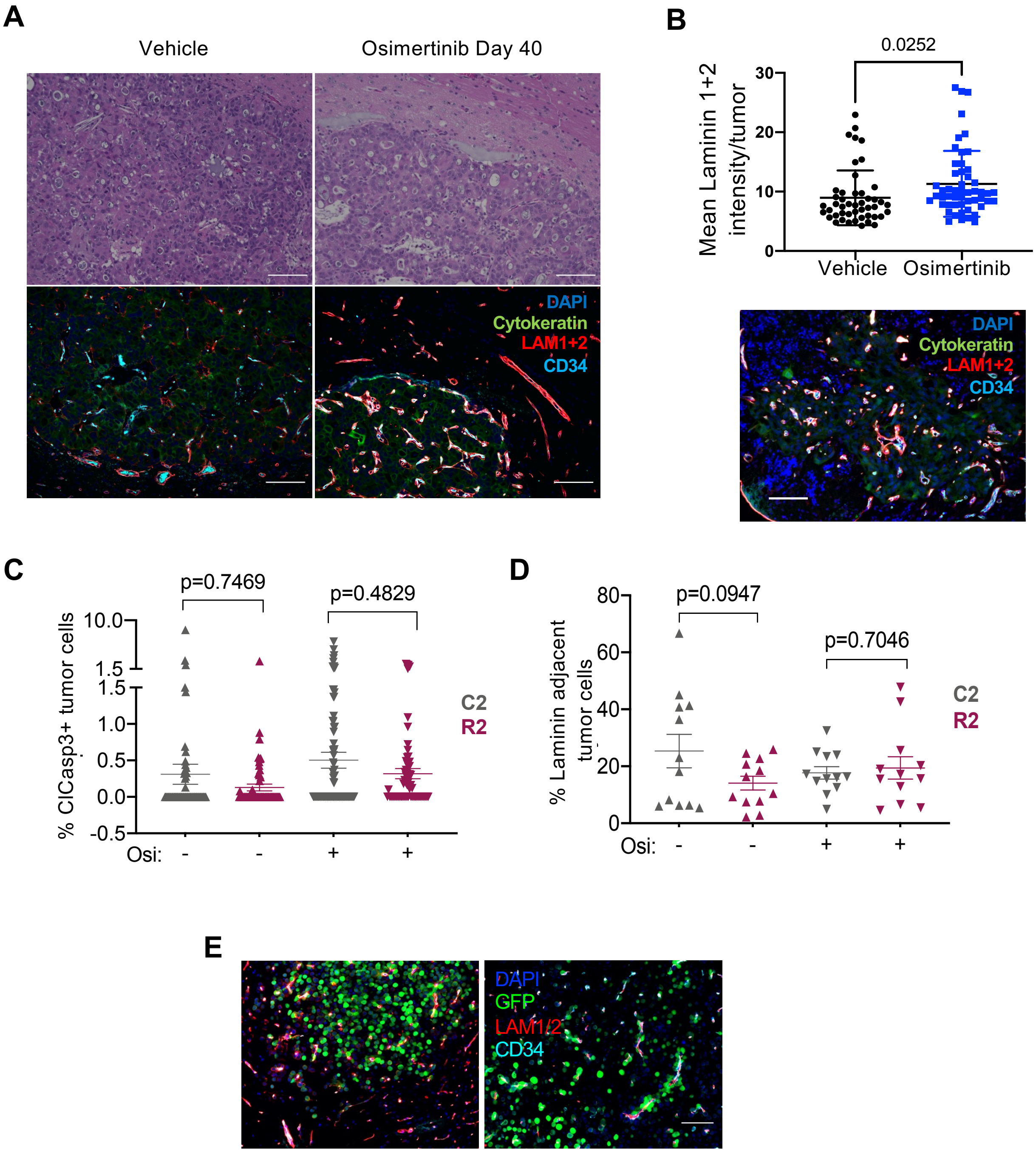

Supplementary Figure 4

Supplementary Figure 4 (related to Figure 5):

(A) Representative images of H&E (top) and IF (bottom) staining of vasculature (CD34; cyan), laminin (red) and nuclei (DAPI; blue) of PDX YU-006 tumor cells (cytokeratin; green) engrafted in the brain and treated with osimertinib for 40 days compared to vehicle-treated tumors. Scale bar indicates 100µm. **(B)** Top: Quantification of laminin intensity in established H1975 brain metastasis treated with vehicle or osimertinib after for 41 days. Vehicle (N=47 images from 3 mice); osimertinib-treated (N=52 images from 6 mice). Data are presented as mean values +/- SD. P-value was calculated by t-test (two-sided). Bottom: Representative IF image of H1975 brain metastasis (GFP; green), laminin (red), vasculature (CD34; cyan/turquoise) and nuclei (DAPI; blue) at day 41 post–osimertinib treatment. Scale bar indicates 100µm. **(C)** % Cleaved Caspase 3 (ClCasp3)-positive brain metastatic cells in vivo was quantified as in Figure 5E. C2 vehicle, N=67; R2 vehicle, N=70; C2 osimertinib, N=94; R2 osimertinib N=65. Images are from 3 animals per group. Data presented as mean values +/- SEM. P-values for IF quantification calculated by Mann-Whitney (two-sided) **(D)** The number of GFP positive R2 tumor cells adjacent to laminin positive micro-vessels at Early timepoint were quantified as in Figure 5C. Data presented as mean values +/- SEM. N= 12 images of 3 animals per group**.** P-values were calculated by Mann-Whitney (two-sided). **(E)** Representative images of R2 brain metastasis (N=12 images from 3 mice) treated with vehicle or osimertinib as in Figure 5B. Immunofluorescent (IF) staining for nuclei (DAPI; blue), tumor cells (GFP; green), laminin (red), and vasculature (CD34; turquoise). Scale bar indicates 100µm.

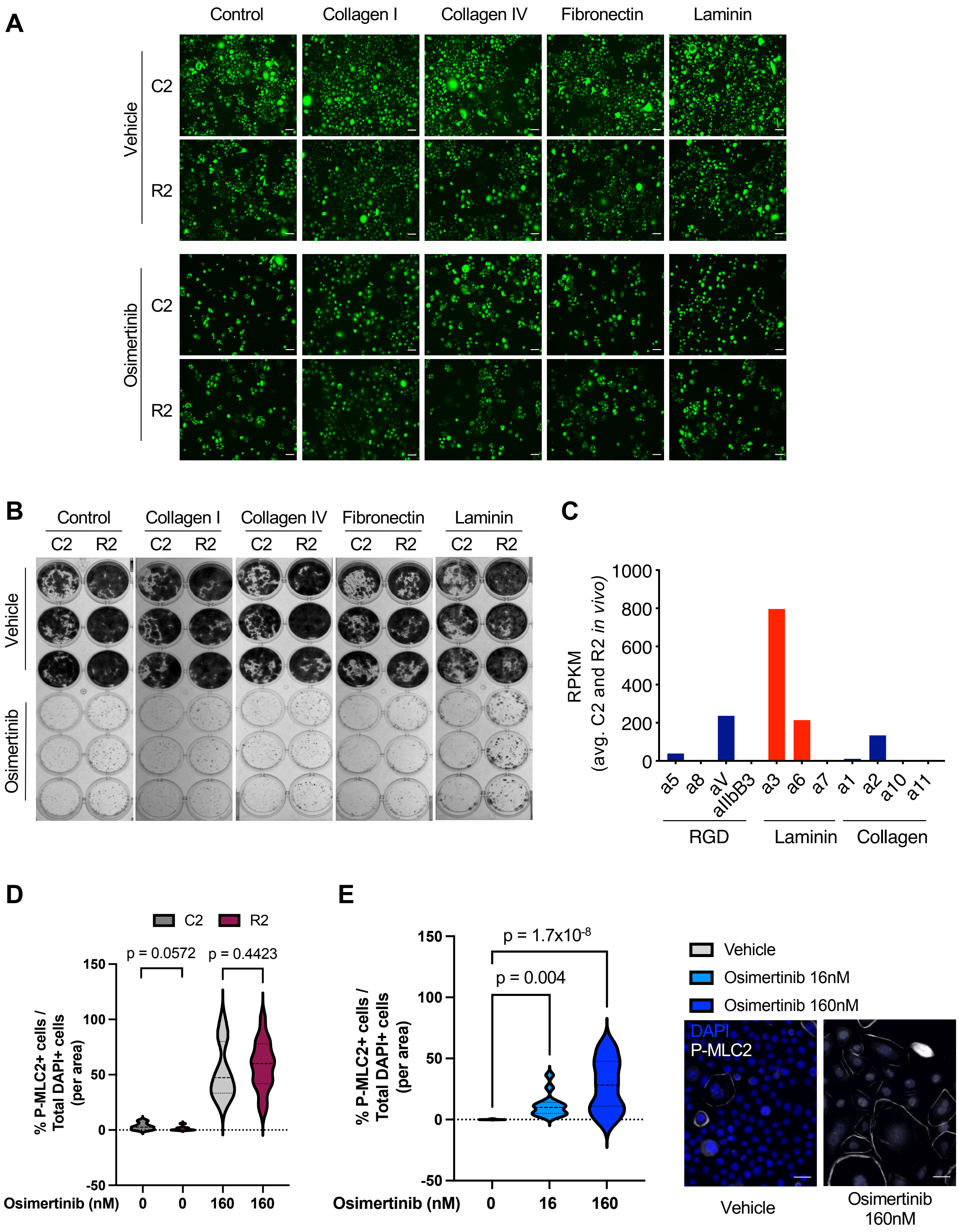

Supplementary Figure 5

Supplementary Figure 5 (related to Figure 6):

(A) Immunofluorescent images of GFP+ C2 and R2 cells plated on the annotated ECM coated plates and treated with vehicle or osimertinib for five days. Images are representative of 3 independent experiments. Scale bar indicates 100µm. **(B)** Representative images of all samples in Figure 6A stained with crystal violet. **(C)** Expression of the indicated α integrin receptor genes in R2 tumor cells in the brain was plotted as reads per kilobase of transcript per million mapped reads (RPKM) from BMX-seq profiled brain metastasis samples form Figure 4A. (**D**) Cells were treated as in Figure 6A, fixed, and stained for P-MLC2 and DAPI. Cells with membrane staining of P-MLC2 were plotted as percentage of total number of cells (DAPI+). Data presented as violin plot (mean and quartiles shown with dotted lines). Statistics were calculated with Mann Whitney (two-sided). N=15 fields of view. **(E)** R2 cells were plated in laminin-coated plates and treated with increasing concentrations of osimertinib, fixed and stained for P-MLC2 and DAPI. Quantification was performed as in (D). Representative immunofluorescent images of P-MLC2. Scale bar = 200µm. Data presented as violin plot (mean and quartiles shown with dotted lines). Statistics were calculated with ANOVA Kruskall wallis. N=15 fields of view, except osimertinib-treated (16nM) N=14 fields of view.

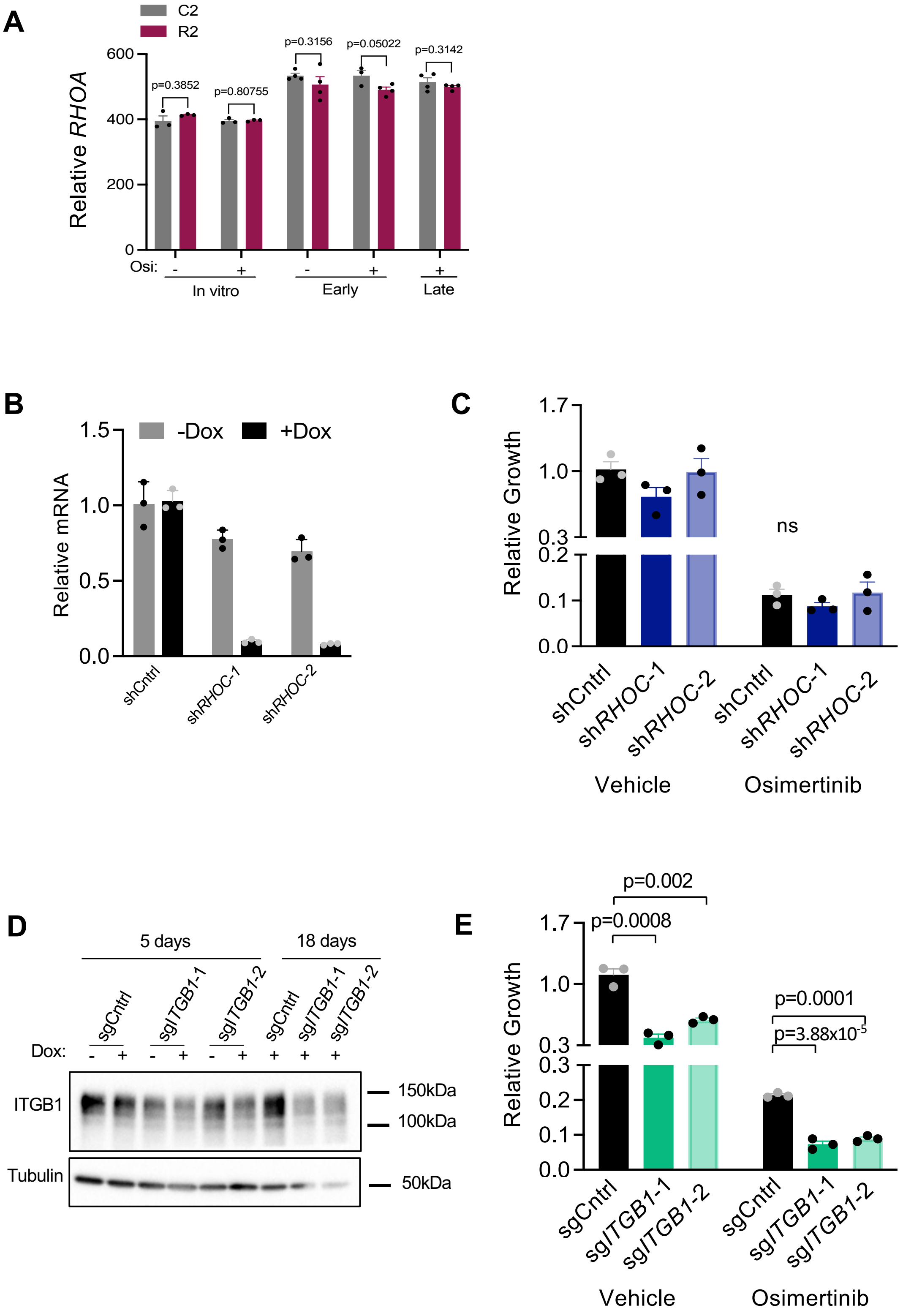

Supplementary Figure 6

Supplementary Figure 7 (related to Figure 7):

(A) Western bots for cofilin and phosphorylated cofilin (p-Cofilin) were performed on the same samples from Figure 7A. Loading control blots for Cofilin (Tubulin) and p-Cofilin (GAPDH) are the same as Figure 7A. **(B)** C2 or R2 cells were cultured over 18 days in the presence of osimertinib on control or laminin-coated plates. *SRF* mRNA was measured by qPCR, normalized to *HPRT* expression and data presented as mean +/- SD. N=3 technical replicates from 1 experiment. **(C)** R2 cells were cultured over 18 days in the absence or presence of the indicated amount of osimertinib on laminin coated plates. Lysates were then subjected to western blotting for the indicated proteins. A representative of two independent experiments is shown. **(D)** R2 cells expressing the indicated shRNAs were cultured on laminin-coated plates with Dox, serum starved for 12 hours, and treated with 10% fetal bovine serum (FBS) and osimertinib for 0, 0.25, and 6 hours. Lysates were then subjected to western blotting for the indicated proteins. A representative of two independent experiments is shown. **(E)** Western blot SRF in R2 cells expressing Dox-inducible single guide RNAs (sgRNAs) against *ROSA26* (sgCntrl) or one of two independent sgRNAs targeting *SRF.* Cells were treated with Dox for 5 days before harvest. A representative of two experiments is shown. **(F)** R2 cells with the indicated sgRNAs were pre-treated with Dox, cultured on laminin coated plates and treated with vehicle or Osimertinib. Relative tumor cell growth was measured as in Figure 6A. All values are normalized to R2 sgCntrl vehicle treated. N=3 samples. Data are presented as mean values +/- SD and p-values calculated by t-test (two-sided). Data is representative of 2 independent experiments. **(G-H)** Relative mean expression (RPKM) of *FOSL1* and *KRT13* were plotted for single cells collected from NSCLC patients as in Supplementary Figure 2E. TN=treatment naïve patients. PR=Patients with partial responses. PD=patients with progressive disease. P-value calculated by Welch's t-test (two-sided). A similar result for *SERPINE1* was reported in [24].

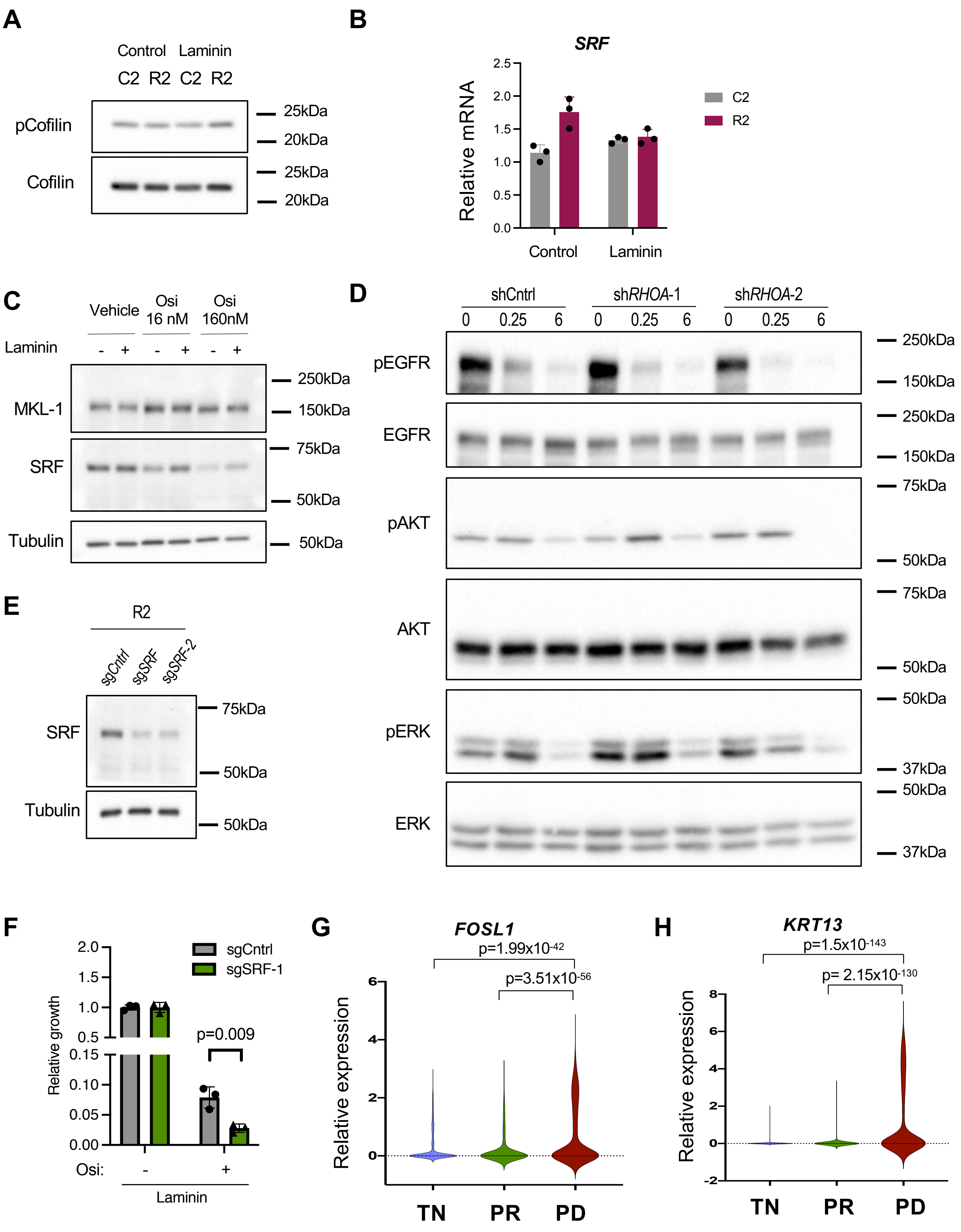

Supplementary Figure 7

Supplementary Figure 7 (related to Figure 7):

(A) Western bots for cofilin and phosphorylated cofilin (p-Cofilin) were performed on the same samples from Figure 7A. Loading control blots for Cofilin (Tubulin) and p-Cofilin (GAPDH) are the same as Figure 7A. **(B)** C2 or R2 cells were cultured over 18 days in the presence of osimertinib on control or laminin-coated plates. *SRF* mRNA was measured by qPCR, normalized to *HPRT* expression and data presented as mean +/- SD. P-value calculated by Wech's t-test (two-sided). N=3 technical replicates from 1 experiment. **(C)** R2 cells were cultured over 18 days in the absence or presence of the indicated amount of osimertinib on laminin coated plates. Lysates were then subjected to western blotting for the indicated proteins. A representative of two independent experiments is shown. **(D)** R2 cells expressing the indicated shRNAs were cultured on laminin-coated plates with Dox, serum starved for 12 hours, and treated with 10% fetal bovine serum (FBS) and osimertinib for 0, 0.25, and 6 hours. Lysates were then subjected to western blotting for the indicated proteins. A representative of two independent experiments is shown. **(E)** Western blot SRF in R2 cells expressing Dox-inducible single guide RNAs (sgRNAs) against *ROSA26* (sgCntrl) or one of two independent sgRNAs targeting *SRF.* Cells were treated with Dox for 5 days before harvest. A representative of two experiments is shown. **(F)** R2 cells with the indicated sgRNAs were pre-treated with Dox, cultured on laminin coated plates and treated with vehicle or Osimertinib. Relative tumor cell growth was measured as in Figure 6A. All values are normalized to R2 sgCntrl vehicle treated. N=3 samples. Data are presented as mean values +/- SD and p-values calculated by t-test (two-sided). Data is representative of 2 independent experiments. **(G-H)** Relative mean expression (RPKM) of *FOSL1* and *KRT13* were plotted for single cells collected from NSCLC patients as in Supplementary Figure 2E. TN=treatment naïve patients. PR=Patients with partial responses. PD=patients with progressive disease. P-value calculated by Welch's t-test (two-sided). A similar result for *SERPINE1* was reported in [24].

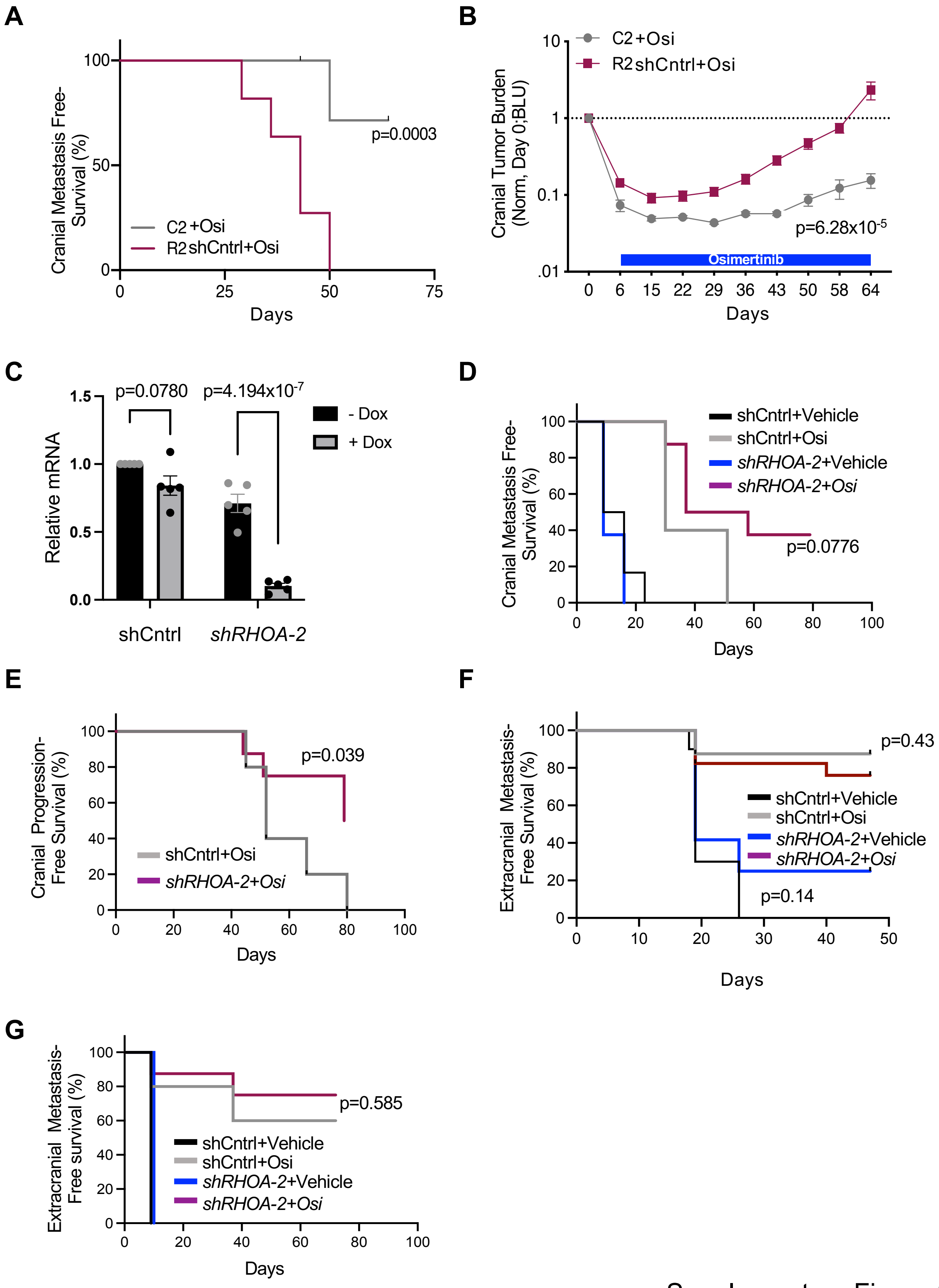

Supplementary Figure 8

Supplementary Figure 8 (Related to Figure 8)

(A) Kaplan-Meier analysis for brain metastasis-free survival of mice following intracardiac injection of C2 or R2 shCntrl cells. Animals were treated with osimertinib starting six days after tumor cell injection. Incidence of metastasis was detected by BLI imaging. N=8 animals for C2, and N=11 for R2 shCnrtl. P-value calculated by log-rank test. **(B)** Cranial tumor burden of mice from (A). Tumor burden is measured by cranial or extracranial BLI values, which are then normalized to measurements on Day 0 of injection for each animal. N=8 animals for C2, and N=11 for R2 shCntrl. Data presented as mean +/- SEM. P-values for AUC calculated by Mann-Whitney (two-sided). **(C)** H1975 cells expressing doxycycline (Dox) inducible short hairpins RNAs (shRNAs) against a control sequence (shCntrl) or shRNAs targeting *RHOA* (sh*RHOA*-2) were cultured in the absence or presence of Dox. *RHOA* was measured by qPCR, normalized to *HPRT* expression, and data was normalized to shCntrl not treated with Dox. N=5 independent experiments. Data presented as mean values +/- SEM. P-value were calculated using 2-way ANOVA. **(D)** Kaplan-Meier analysis of brain metastasis incidence following intracardiac injection of H1975 shCntrl or H1975 sh*RHOA*-2 cells into mice that were maintained on a Dox feed diet and treated with either vehicle or osimertinib starting 2-3 days after injection. Brain metastasis was detected by BLI. N = 5 for shCntrl and N=8 animals for sh*RHOA*-2 . **(E)** Kaplan-Meier analysis of brain metastasis progression for animals in (D). Progression was defined as when the cranial tumor burden of treated mice reaches 200% of the pre-treatment cranial tumor burden as previously described [51]. **(F)** Kaplan-Meier analysis for extra-cranial metastasis-free survival of mice in Figure 7A. **(G)** Kaplan-Meier analysis for metastasis-free survival of mice in (E). For D, E, F, and G, P-values were calculated by log-rank test.

Chr	Start	End	Ref	Alt	Func.refGene	Gene.refGene	AAChange.refGene	Revel score
14	96782965	96782965	T	C	exonic	ATG2B	ATG2B:NM_018036:exon21:c.A3266G:p.N1089S	0.094
14	51372118	51372118	C	T	exonic	PYGL	PYGL:NM_001163940:exon19:c.G2434A:p.G812R, PYGL:NM_002863:exon20:c.G2536A:p.G846R	0.33
14	105930406	105930406	G	A	exonic	MTA1	MTA1:NM_001203258:exon13:c.G1114A:p.V372I, MTA1:NM_004689:exon13:c.G1114A:p.V372I	0.022
14	93118038	93118038	A	G	exonic	RIN3	RIN3:NM_001319987:exon5:c.A419G:p.H140R,RIN3:NM_024832:exon6:c.A644G:p.H215R	0.018
14	93154538	93154540	GGC	-	exonic	RIN3	RIN3:NM_001319987:exon9:c.2674_2676del:p.G897Sfs*14,RIN3:NM_024832:exon10:c.2899_2901del:p.G972Sfs*14	n/a

Supplementary Table 1: Summary of whole exome sequencing comparing R2 to C2 cells. Listed are potential point mutations in R2 cells.

Antibody	Company	Catalog #	Source	Clone	Dilution	Application
pEGFR	CST	3777	Rabbit mAb	D7A5	1:100	IHC
COLIV	Millipore	AB756	Rabbit		1:100	IF
LAM1/2	Abcam	Ab7463	Rabbit		1:100	IF
CD34	Abcam	Ab8158	Rat mAb	MEC 14.7	1:100	IF
pHH3	CST	9701	Rabbit		1:800	IF
CC3, Cleaved Caspase-3	CST	9661	Rabbit		1:50	IF
PanCytokeratin	E-bioscience	53-9003-8	Mouse mAb	AE1/AE3	1:100	IF
pMLC2	CST	3674	Rabbit		1:200	IF
Alexa Fluor® 647 AffiniPure Donkey Anti-Rabbit IgG (H+L)	Jackson ImmunoResearch	711-605-152	Donkey		1:200	IF
Rhodamine Red™-X (RRX) AffiniPure Donkey Anti-Rat IgG (H+L)	Jackson ImmunoResearch	712-295-153	Donkey		1:200	IF
Alexa Fluor® 488 AffiniPure Donkey Anti-Mouse IgG (H+L)	Jackson ImmunoResearch	715-545-151	Donkey		1:200	IF
ITGB1	CST	9699	Rabbit mAb	D2E5	1:2000	WB
Tubulin	Sigma	T5168	Mouse mAb	B-5-1-2	1:3000	WB
GAPDH	CST	2118	Rabbit mAb	14C10	1:1000	WB
pCofilin	CST	3313	Rabbit mAb	77G2	1:1000	WB
Cofilin	CST	5175	Rabbit mAb	D3F9	1:1000	WB
pEGFR	CST	3777	Rabbit mAb	D7A5	1:1000	WB
EGFR	CST	2232	Rabbit		1:1000	WB
pAKT	CST	4056	Rabbit mAb	244F9	1:500	WB
AKT	CST	4691	Rabbit mAb	C67E7	1:1000	WB
pERK	CST	4370	Rabbit mAb	D13.14.4E	1:1000	WB
SRF	CST	5147	Rabbit mAb	D71A9	1:1000	WB
p-YAP	Abcam	76252	Rabbit mAb	EP1675Y	1:1000	WB
YAP	CST	8418	Rabbit mAb	D24E4	1:1000	WB
MKL-1	Bethyl	A302-201A-M	Rabbit	polyclonal	1:1000	WB
Anti-mouse HRP	Thermo Scientific	31437	Rabbit	polyclonal	1:5000	WB
Anti-rabbit HRP	Thermo Scientific	31458	Donkey	polyclonal	1:5000	WB

Supplementary Table 2: List of antibodies and dilutions

Gene	Species	qPCR Assay ID	Company
HPRT1	Human	Hs99999909_m1	Thermo Fischer Scientific
RHOA	Human	Hs01051295_m1	Thermo Fischer Scientific
RAP1A	Human	Hs05011994_s1	Thermo Fischer Scientific
RHOC	Human	Hs00733980_m1	Thermo Fischer Scientific
KRT13	Human	Hs00999762_m1	Thermo Fischer Scientific
FOSL1	Human	Hs04187685_m1	Thermo Fischer Scientific
SERPINE1	Human	Hs00167155_m1	Thermo Fischer Scientific
SRF	Human	Hs05484803_s1	Thermo Fischer Scientific

Supplementary Table 3: List of qPCR probes

Designation	Target sequence
sgRosa26	GTCGCTTCTCGATTATGGGC
sgITGB1-1	GTGGCGCGTGCAGGTAAGCT
sgITGB1-5	TCATCACATCGTGCAGAAGT
sgSRF-1	ACCAGGTGTCGGAGTCTGAC
sgSRF-2	CCATGCAAGTCAGCAGCGGC
shCntrl	AGGCTATTACTCACCGTATTAT
shRHOA-1	AACACACCAGGCGCTAATTCAA
shRHOA-2	AGTACATGGAGTGTTCAGCAAA
shRHOC-1	ACTACTGTCTTTGAGAACTATA
shRHOC-2	ACGAAAGAAGCTGGTGATCGTT

Supplementary Table 4: sgRNA and shRNAs sequences
