# Supplementary material for: Brain metastatic outgrowth and osimertinib resistance are potentiated by RhoA in EGFR-mutant lung cancer
Source: Nat Commun. 2022 Dec 12;13:7690. doi: 10.1038/s41467-022-34889-z (PMC9744876; doi:10.1038/s41467-022-34889-z)
Supplement: Supplementary file 5 — Description of Additional Supplementary Files [file 41467_2022_34889_MOESM5_ESM.docx]

**Inventory of supporting information:**

**Supplementary Data 1:** scRNA-seq analysis of R2 an C2 cells. Genes that distinguish different PC9 sub-populations are provided.

**Supplementary Data 2:** MetRes gene signature. List of genes that distinguish sub-populations 1,5, and 8.

**Supplementary Data 3:** BMX-seq tumor specific genes that are expressed in R2 and C2 tumors *in vivo.* A column Key is described below:

anova padj: ANOVA adjusted p value (stepup) for all possible comparisons

R2: brain metastasis from R2 cells

C2: brain metastasis from C2 cells

o3: brain metastasis treated with osimertinib for 3 days

oL: brain metastasis treated with osimertinib for 28-56 days

v3: brain metastasis vehicle treated 3 days

vL: brain metastasis vehicle treated 28-56 days

o2D: Cells in culture treated with osimertinib for 24 hours

V2D: Cells in culture treated with vehicle for 24 hour

Bold=comparisons used to define the gene list

Example: R2o3.C2o3: R2 brain metastasis versus C2 brain metastasis; treated with osimertinib for 3 days. R2oL.C2oL: R2 brain metastasis versus C2 brain metastasis; treated with osimertinib for 28-56 days

**Source Data File:** Source Data for all Figure panels.
